# Plant intraspecific functional trait variation is related to within‐habitat heterogeneity and genetic diversity in *Trifolium montanum* L.

**DOI:** 10.1002/ece3.6255

**Published:** 2020-04-16

**Authors:** Kevin Karbstein, Kathleen Prinz, Frank Hellwig, Christine Römermann

**Affiliations:** ^1^ Institute of Ecology and Systematics Friedrich‐Schiller‐University Jena Jena Germany; ^2^ German Centre for Integrative Biodiversity Research (iDiv) Halle‐Jena‐Leipzig Leipzig Germany; ^3^ Present address: Department of Systematics, Biodiversity and Evolution of Plants (with Herbarium) University of Goettingen Albrecht‐von‐Haller Institute for Plant Sciences Goettingen Germany; ^4^ Present address: Landschaftspflegeverband Suedharz/Kyffhaeuser e.V. Nordhausen Germany

**Keywords:** (semi‐)dry grasslands, environmental heterogeneity, functional traits, intraspecific functional trait variation (iFD_CV_), mountain clover, population genetics

## Abstract

Intraspecific trait variation (ITV), based on available genetic diversity, is one of the major means plant populations can respond to environmental variability. The study of functional trait variation and diversity has become popular in ecological research, for example, as a proxy for plant performance influencing fitness. Up to now, it is unclear which aspects of intraspecific functional trait variation (iFD_CV_) can be attributed to the environment or genetics under natural conditions. Here, we examined 260 individuals from 13 locations of the rare (semi‐)dry calcareous grassland species *Trifolium montanum* L. in terms of iFD_CV_, within‐habitat heterogeneity, and genetic diversity. The iFD_CV_ was assessed by measuring functional traits (releasing height, biomass, leaf area, specific leaf area, leaf dry matter content, F_v_/F_m_, performance index, stomatal pore surface, and stomatal pore area index). Abiotic within‐habitat heterogeneity was derived from altitude, slope exposure, slope, leaf area index, soil depth, and further soil factors. Based on microsatellites, we calculated expected heterozygosity (H_e_) because it best‐explained, among other indices, iFD_CV_. We performed multiple linear regression models quantifying relationships among iFD_CV_, abiotic within‐habitat heterogeneity and genetic diversity, and also between separate functional traits and abiotic within‐habitat heterogeneity or genetic diversity. We found that abiotic within‐habitat heterogeneity influenced iFD_CV_ twice as strong compared to genetic diversity. Both aspects together explained 77% of variation in iFD_CV_ ($R_{\text{adj}}^{2}$ = .77, *F*_2, 10_ = 21.66, *p* < .001). The majority of functional traits (releasing height, biomass, specific leaf area, leaf dry matter content, F_v_/F_m_, and performance index) were related to abiotic habitat conditions indicating responses to environmental heterogeneity. In contrast, only morphology‐related functional traits (releasing height, biomass, and leaf area) were related to genetics. Our results suggest that both within‐habitat heterogeneity and genetic diversity affect iFD_CV_ and are thus crucial to consider when aiming to understand or predict changes of plant species performance under changing environmental conditions.

## Graphical abstract

Up to now, it is unclear which aspect of intraspecific functional trait variation (ITV) can be attributed to the environment or to genetics under natural and present environmental conditions. Here, we used data from an extensive field study (260 individuals from 13 locations) on the (semi-)dry calcareous grassland species Trifolium montanum (mountain clover). We demonstrated that abiotic within-habitat heterogeneity had twice as much impact as genetic diversity on ITV explaining together 77% of variation.

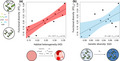

## 1 INTRODUCTION

Functional traits are morphological, (eco‐)physiological, and also reproductive traits that impact an individual's growth, reproduction, and survival and thus influence plant fitness indirectly but also directly (Nock, Vogt, & Beisner, 2016; Violle et al., 2007). Gained from direct measurements or databases (e.g., TRY, Kattge et al., 2011; Kattge et al., 2020), they have been used to investigate responses of populations, species, communities, and ecosystems to the environment (e.g., land use, climate; Bernhardt‐Römermann, Gray, et al., 2011; Bucher et al., 2016; Díaz & Cabido, 2001; Gratani, 2014; König et al., 2018; Nicotra et al., 2010; Römermann, Bernhardt‐Römermann, Kleyer, & Poschlod, 2009; Violle et al., 2007).

Intraspecific trait variation (ITV) depends on the available phenotypic trait plasticity of individuals within a population. Phenotypic plasticity, that is, the phenotypic variation expressed by a single genotype under different environmental conditions (Hufford & Mazer, 2003; Nicotra et al., 2010; Sultan, 2000), might be one of the most important mechanisms for plants in reacting to environmental changes (e.g., land use, climate change; Agrawal, 2001; Arnold, Kruuk, & Nicotra, 2019; Gratani, 2014; Via et al., 1995). In general, environment and genetics can generate ITV (de Bello et al., 2011; Violle et al., 2012). The complex relationships between population‐based ITV of functional traits and environmental heterogeneity of the habitats where traits of populations have been investigated, however, have not yet received much attention.

Genetic diversity is considered to be fundamental for population fitness and evolutionary processes, and influences adaptive potential of a species with respect to environmental changes, competitors or pathogens (Arnold et al., 2019; Boulding, 2008; Chevin & Hoffmann, 2017; Karbstein, Tomasello, & Prinz, 2019; Lande, 2009; Nicotra et al., 2010; Reed & Frankham, 2003). Relationships between genetic diversity and “traditional” fitness parameters (e.g., number of flowers, seeds and fruits, and seed weight) or life‐history traits have been investigated intensely both within and across species (Freeland, Kirk, & Petersen, 2011; Leimu, Mutikainen, Koricheva, & Fischer, 2006; Reed & Frankham, 2003; Reisch & Bernhardt‐Römermann, 2014). In some model plants, functional trait plasticity related to morphology (e.g., plant height), ecophysiology (e.g., water‐use efficiency), and life history (e.g., flowering time and seed traits) was found to be under genetic control (Ackerly et al., 2000; Hughes, Soppe, & Albani, 2019; Locascio, Lucchin, & Varotto, 2009; Thornsberry et al., 2001). Moreover, studies also indicate phenotypic and genetic connections, and also, more explicitly, correlations between phenotypic traits and genetic variation (Csilléry et al., 2020; Karbstein et al., 2019; Waitt & Levin, 1998). Waitt and Levin (1998) impressively showed positive correlations between the phenotypic variation of morphology‐related functional traits and genetic variation in several species from different plant families. Nevertheless, trait variation, if it is for example entirely plastic, does not necessarily coincide with genetic variation (see also Chevin & Hoffmann, 2017). Relationships between intraspecific functional trait variation and genetic diversity at population‐level and under natural environmental conditions remain poorly understood.

Considerable functional differences may provide improved resource partitioning due to differences in niche exploitation and/or a more flexible response to environmental changes (see Bucher et al., 2016; MacArthur & Wilson, 1967; Schweiger et al., 2018; Simpson, 1949; Violle et al., 2012). Within populations, increased ITV based on within‐habitat heterogeneity should be able to increase adaptability with positive consequences for growth, reproduction, and survival. Environmental heterogeneity within habitats may thus lead to an increased number of different functional phenotypes and thus enhances ITV. Moreover, habitat heterogeneity is expected to influence the genotypic range of variation within a habitat: Variable environments can exert different selective pressures generating genetic heterogeneity (Gratani, 2014; Linhardt & Grant, 1996; Sakaguchi et al., 2019). Nevertheless, within a habitat of a population, environmental differences are usually lower and gene flow more frequently (for example due to missing geographical barriers) than between habitats across larger scales. Within‐habitat heterogeneity might also enhance the occurrence of different genotypes due to different resource exploitation possibilities, increasing genetic variation (see, e.g., Agashe & Bolnick, 2010; Reusch, Ehlers, Hämmerli, & Worm, 2005). Therefore, within‐habitat heterogeneity affects ITV directly and genetic diversity indirectly. However, ITV, within‐habitat heterogeneity, and genetic diversity may interact in complex ways under natural environmental conditions. For example, plasticity of traits (generating ITV) is able to influence the selective effect of within‐habitat heterogeneity on genetic diversity, whereas connectivity and dispersal (gene flow) among habitats can affect selection on genetic diversity (Ghalambor, McKay, Carroll, & Reznick, 2007; Linhardt & Grant, 1996; Reisch & Schmid, 2019; Vellend & Geber, 2005).

**FIGURE 1:**
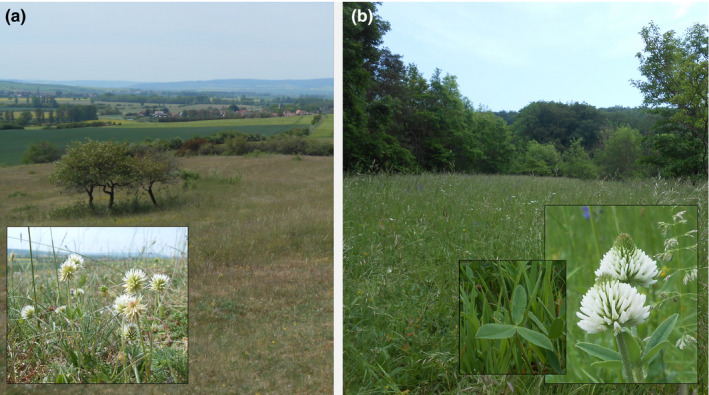
*Trifolium montanum* in different habitats. (a) Location Bottendorf (Bo): Small *T. montanum* individuals grow on continental‐dry grasslands. (b) Location Jena‐Wogau (Wo): *T. montanum* individuals inhabit semi‐dry *Bromus erectus* grasslands along the forest margin. Mountain clover is characterized by denticulate leaflets with silky abaxial leaf surfaces (see a and b). Image source: Karbstein (2016)

In this study, we aim to investigate the relative effects of abiotic within‐habitat heterogeneity and genetic diversity on intraspecific trait variation (ITV). Investigations are based on 260 individuals from 13 Central European populations of the rare (semi‐)dry calcareous grassland species *Trifolium montanum* L. (mountain clover; Figure 1). We addressed the following questions: Is ITV related to abiotic within‐habitat heterogeneity and/or genetic diversity? If so, to what extent is ITV explained by either aspect? Which functional traits are related to abiotic within‐habitat heterogeneity and/or genetic diversity?

## 2 MATERIALS AND METHODS

### 2.1 Model species

**FIGURE 2:**
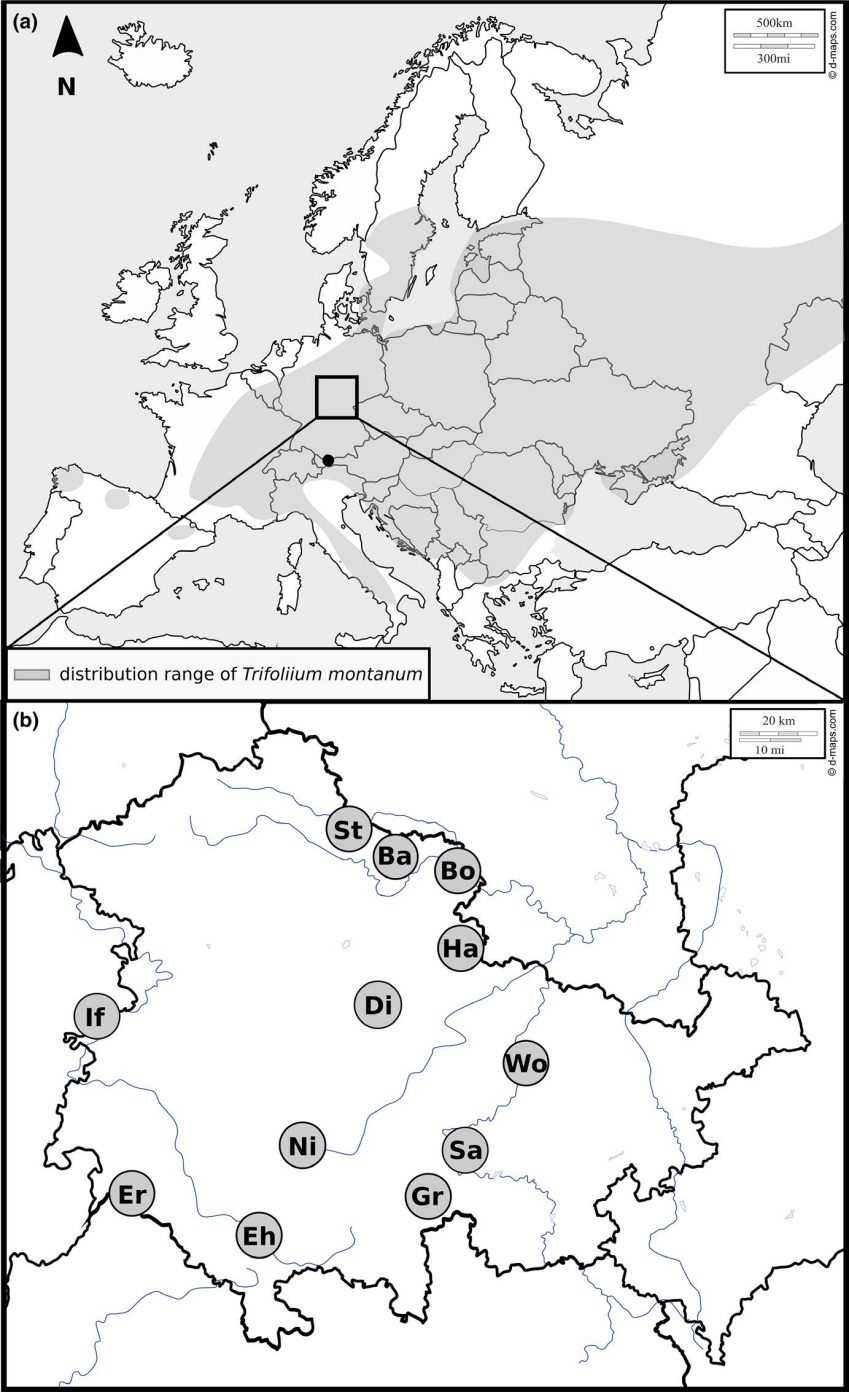
(a) Distribution range of *Trifolium montanum* in Europe (light gray) according to Meusel and Jäger (1998). The black square indicates the sampling area in Central Germany. The black dot represents the sampling location in Austria (“KW”). (b) Sampling scheme of the present study in Central Germany (see Table 1 for abbreviations and detailed information). Black circles represent sampling locations. Within circles, location abbreviations are given. Black lines indicate borders of the German Federal States (focus on Thuringia). Basic geographical maps were downloaded from d‐maps.com

*Trifolium montanum* L. (Fabaceae) is a perennial herb of extensively used, calcareous grasslands distributed in Europe and Western Russia (Figures 1 and 2; GBIF Secretariat, 2017; Hahn, Kettle, Ghazoul, Hennig, & Pluess, 2013; Schleuning & Matthies, 2008; Schleuning, Niggemann, Becker, & Matthies, 2009). Populations usually inhabit (semi‐)dry grasslands (Jäger, 2011), but they also occur along with shrub and forest margins. In Central Europe, the species is quite rare because of  degradation and fragmentation of (semi‐)dry grasslands (Garve, 2004; Schleuning & Matthies, 2008; Schleuning et al., 2009). *Trifolium montanum* is diploid with 2n = 16 (Rice et al., 2015).

### 2.2 Study locations and sampling

We focused on 13 locations in total, from which 12 are situated in Germany and, to cover a larger range of environmental conditions, one in Austria (Table 1). *Trifolium montanum* populations at the 13 different locations are independent of each other: The average distance of mating events in this species is quite low (10 m), and thus, the majority of mating events occur on small distances (pollen dispersal up to a distance of 324 m possible; Matter, Kettle, Ghazoul, Hahn, & Pluess, 2013). This indicates a reduced potential for pollen‐mediated long‐distance dispersal. For two *Trifolium* species, the estimated long‐distance dispersal via seeds is also estimated to be only six to 10 m (Vittoz & Engler, 2007). We estimated an average distance among study locations of ca. 133 km (80 km without the distant location KW) and standard deviation of 124 km (41 km without the distant location KW, see Table S3 for details and the applied “geosphere” r package vers. 1.5‐5 (Hijmans, 2016) using the Vincenty ellipsoid method). Distances are thus too large for direct pollen exchange or seed dispersal. There is only the possibility for direct gene flow between locations Bo and Ha, and Ba and St. However, locations are separated by large agrarian areas (particularly Ha), forests, and roads, minimizing the probability of pollen exchange or seed‐mediated long‐distance dispersal and thus the potential of gene flow. *Trifolium montanum* is consumed by grazing animals, but Bo was grazed by sheep and goats, St by cattle, and the management of Ha is unknown, suggesting rather very local grazer movements instead of habitat connection via transhumance.

**TABLE 1:** Location, date of sampling, latitude (lat. (N), longitude (long. (E.), and mean coefficients (with standard errors in brackets) for variation of intraspecific functional trait variation (iFD_CV_), abiotic within‐habitat heterogeneity (HD), and mean genetic diversity (GD; H_e_) of 13 *Trifolium montanum* populations

Location	Date	Lat. (*N*)	Long. (E)	iFD_CV_	HD	GD
Riezlern (KW)	17.07.2015	47.361,036	10.173,825	0.173 (±0.044)	0.143 (±0.044)	0.597 (±0.083)
Bottendorf (Bo)	22.05.2016	51.316,042	11.396,525	0.228 (±0.058)	0.303 (±0.091)	0.612 (±0.072)
Hardisleben (Ha)	25.05.2016	51.162,917	11.446,789	0.205 (±0.055)	0.235 (±0.056)	0.630 (±0.098)
Jena‐Wogau (Wo)	29.05.2016	50.924,306	11.665,083	0.210 (±0.054)	0.257 (±0.064)	0.654 (±0.080)
Bad Frankenhausen (Ba)	31.05.2016	51.367,267	11.103,056	0.196 (±0.048)	0.244 (±0.054)	0.666 (±0.060)
Steinthaleben (St)	05.06.2016	51.409,550	11.004,850	0.265 (±0.076)	0.357 (±0.102)	0.686 (±0.060)
Saalfeld (Sa)	08.06.2016	50.631,003	11.383,729	0.242 (±0.061)	0.325 (±0.107)	0.637 (±0.073)
Ifta (If)	12.06.2016	51.086,633	10.148,017	0.184 (±0.044)	0.150 (±0.042)	0.678 (±0.067)
Niederwillingen (Ni)	15.06.2016	50.776,294	11.027,711	0.204 (±0.049)	0.169 (±0.053)	0.661 (±0.085)
Dielsdorf (Di)	19.06.2016	51.095,233	11.188,406	0.202 (±0.056)	0.295 (±0.111)	0.647 (±0.072)
Erbenhausen (Er)	23.06.2016	50.565,556	10.157,383	0.224 (±0.043)	0.346 (±0.097)	0.658 (±0.078)
Großneundorf (Gr)	28.06.2016	50.532,456	11.294,961	0.149 (±0.036)	0.151 (±0.044)	0.570 (±0.090)
Ehrenberg (Eh)	29.06.2016	50.478,583	10.665,786	0.153 (±0.031)	0.193 (±0.064)	0.595 (±0.084)

From each location, we collected 20 individuals of a *T. montanum* population, totaling 260 individuals. We attempted to distribute individual sampling points equally within a habitat. Fieldwork was done in July 2015 (Austria) and from May to June 2016 (Germany).

### 2.3 Functional traits—measurements and ecological meaning

As traits change with the season (Bucher et al., 2019; Römermann, Bucher, Hahn, & Bernhardt‐Römermann, 2016), we started sampling in lowlands and finished sampling in higher altitudes (compare also Tautenhahn, Grün‐Wenzel, Jung, Higgins, & Römermann, 2019). To ensure the comparability of functional traits among populations, we only sampled flowering and early fruiting individuals to control for phenology (Römermann et al., 2016). All functional traits were measured on 20 different individuals per population, and all leaf functional traits were measured on two leaves per individual.

In the field, we measured F_v_/F_m_ and PI on absorption basis after 30 min by high intensity focused LED (3,500 µmol/m^2^ * s^−1^ intensity and wavelength peak 627 nm) with Pocket PEA, preparing leaves according to the manufacturer's instructions (Hansatech Instruments Ltd., King´s Lynn, England; Strasser, Srivastava, & Tsimilli‐Michael, 2000; Strasser, Tsimilli‐Michael, & Srivastava, 2004). The ratio of variable fluorescence to maximal fluorescence (F_v_/F_m_) is related to the efficiency of PS II electron transport and indicates abiotic and/or biotic stress due to photoinhibition (Butler & Kitajima, 1975; Griffin, Epstein, & Boelman, 2013; Maxwell & Johnson, 2000; Paillotin, 1976). The performance index (PI) represents the photosynthetic performance of a chlorophyll molecule, the vitality of the plant, and its ability to resist constraints from outside (Bucher, Bernhardt–Römermann, & Römermann, 2018; Clark, Landolt, Bucher, & Strasser, 2000; Strasser et al., 2000). We also determined releasing height (RH) as the shortest distance between the ground and the highest flower head [m] (Cornelissen et al., 2003), and cut total fresh aboveground biomass directly above taproot.

In the laboratory, we weighed total dry aboveground biomass (AGB) per individual [g]. We determined the weight of two fresh leaves per individual, conducted leaf scans, and calculated leaf area (LA) using the “LeafTraits” R package vers. 1.0 (Bernhardt‐Römermann, unpubl. data). RH and AGB are related to competitive ability (Chen et al., 2011; Cornelissen et al., 2003; Gaudet & Keddy, 1988; Moles et al., 2009). Leaf area has important consequences for light interception, carbon exchange, and water balance (Díaz et al., 2016; Farquhar, Buckley, & Miller, 2002; Givnish, 1987). Leaf area forms an allometric complex together with RH and AGB due to anatomical and architectural consequences, representing morphology‐related functional traits (Ackerly & Donoghue, 1998; Bartelink, 1997; Cornelissen et al., 2003).

Subsequently, leaves were oven‐dried and weighed again [mg]. We calculated individual mean values for SLA (Cornelissen et al., 2003; Pérez‐Harguindeguy et al., 2013) as the ratio of one‐sided fresh LA [mm^2^] and its oven‐dry mass [mg], and LDMC (Pérez‐Harguindeguy et al., 2013) as the ratio of oven‐dry mass of a leaf [mg] and its water‐saturated fresh mass [g]. Specific leaf area (SLA) tends to be positively correlated with potential relative growth rate, but negatively with leaf dry matter content (LDMC) that represents leaf longevity/robustness (Cornelissen et al., 2003; Pérez‐Harguindeguy et al., 2013; Römermann et al., 2016).

We took stomata imprints using nail polish from two leaves per individual. Utilizing an Olympus CH40 microscope, we counted stomata density at 200× magnification and measured guard cell length and width at 400× magnification. We determined individual mean values of four measurements for stomata density and of eight measurements for stomata length and width measurements of the abaxial leaf surface. We also calculated stomatal pore surface (SPS) as guard cell length [µm] * guard cell width [µm * π * 4^–1^ (Balasooriya et al., 2009) and stomatal pore area index (SPI) as the product of (guard cell length)^2^ [mm^2^] * stomatal density [1/mm^2^] (Sack, Cowan, Jaikumar, & Holbrook, 2003). SPS characterizes stomata size, SPI indicates stomatal conductance, and both traits are known to change along abiotic environmental gradients (Bucher et al., 2016, 2017; Woodward, Lake, & Quick, 2002).

Selected functional traits cover a broad range of plant trait space (see, e.g., Díaz et al., 2016; Gratani, 2014), and many of them are already known to respond to environmental conditions (see, e.g., Cornelissen et al., 2003; Nicotra et al., 2010; Pérez‐Harguindeguy et al., 2013).

### 2.4 Assessment of habitat characteristics

We characterized each location with a maximum of five environmental replicate measurements. The replicates (grid cell 2 m × 2 m) were equally distributed within the local range of each population (20 to 4,600 m^2^, unpubl. data). Therefore, the distance between replicates differed according to habitat sizes. For one location (Eh, see Tables 1 and 2), we reduced the number of replicates to four due to the limited habitat size (~20 m^2^). In total, we analyzed *n* = 64 records.

**TABLE 2:** Coefficients of variation (CV) of particular functional traits (*n* = 260 individuals), abiotic factors (*n* = 64 replicates), and population genetic indices (*n* = 255 individuals) based on nine microsatellite markers (Matter et al., 2012) of 13 *Trifolium montanum* populations. *F*_v_/*F*_m_ and PI measurements are missing at the location Riezlern (KW). Due to data completeness and comparability of iFD_CV_ among populations, we approximated these values by linear regressions

Population	iFD_CV_	N_ind_	CV_RH_	CV_AGB_	CV_LA_	CV_SLA_	CV_LDMC_	CV_Fv/Fm_	CV_PI_	CV_SPS_	CV_SPI_
(KW)		20	0.161	0.429	0.276	0.096	0.050	0.011	0.275	0.105	0.157
(Bo)		20	0.233	0.427	0.305	0.097	0.060	0.023	0.542	0.144	0.225
(Ha)		20	0.199	0.455	0.490	0.101	0.049	0.011	0.212	0.147	0.178
(Wo)		20	0.183	0.493	0.319	0.098	0.102	0.011	0.413	0.100	0.171
(Ba)		20	0.232	0.470	0.278	0.094	0.068	0.015	0.326	0.115	0.170
(St)		20	0.361	0.696	0.496	0.132	0.067	0.019	0.349	0.090	0.176
(Sa)		20	0.324	0.585	0.380	0.108	0.070	0.015	0.334	0.120	0.238
(If)		20	0.167	0.400	0.311	0.087	0.050	0.012	0.310	0.121	0.196
(Ni)		20	0.180	0.407	0.452	0.128	0.064	0.010	0.228	0.141	0.223
(Di)		20	0.172	0.457	0.312	0.121	0.062	0.021	0.458	0.079	0.135
(Er)		20	0.280	0.381	0.373	0.174	0.104	0.011	0.337	0.130	0.230
(Gr)		20	0.138	0.350	0.258	0.091	0.056	0.008	0.210	0.090	0.137
(Eh)		20	0.156	0.307	0.203	0.088	0.074	0.014	0.262	0.121	0.151

	HD	N_rep_	CV_altitude_	CV_slope exposure_	CV_slope_	CV_LAI_	CV_soil depth_	CV_CECpot_	CV_pH_	CV_N_	CV_P_	CV_K_
(KW)		5	0.001	0.000	0.000	0.282	0.294	0.038	0.091	0.129	0.349	0.248
(Bo)		5	0.013	0.895	0.520	0.587	0.350	0.051	0.030	0.206	0.125	0.251
(Ha)		5	0.022	0.373	0.288	0.496	0.322	0.016	0.164	0.102	0.465	0.100
(Wo)		5	0.021	0.566	0.508	0.381	0.223	0.018	0.041	0.172	0.421	0.217
(Ba)		5	0.018	0.000	0.315	0.537	0.408	0.149	0.146	0.298	0.227	0.345
(St)		5	0.011	1.044	0.684	0.437	0.251	0.028	0.048	0.428	0.413	0.226
(Sa)		5	0.013	0.722	0.974	0.621	0.368	0.030	0.007	0.096	0.203	0.218
(If)		5	0.011	0.141	0.276	0.400	0.274	0.012	0.007	0.097	0.086	0.193
(Ni)		5	0.012	0.000	0.516	0.316	0.281	0.117	0.007	0.084	0.132	0.229
(Di)		5	0.012	0.000	1.129	0.342	0.424	0.028	0.048	0.090	0.406	0.471
(Er)		5	0.009	0.639	0.572	0.509	0.438	0.030	0.012	0.156	0.889	0.210
(Gr)		5	0.009	0.124	0.233	0.447	0.266	0.036	0.024	0.044	0.124	0.204
(Eh)		4	0.006	0.000	0.389	0.552	0.334	0.029	0.007	0.071	0.365	0.177

Population	GD	N_ind_	N_A_	P_AP_	H_e_	H_o_	I
(KW)		20	52	5.77	0.597	0.533	1.251
(Bo)		20	63	1.59	0.612	0.604	1.343
(Ha)		20	71	5.63	0.630	0.594	1.450
(Wo)		20	68	4.41	0.654	0.654	1.460
(Ba)		19	53	0.00	0.666	0.560	1.384
(St)		19	63	1.59	0.686	0.662	1.472
(Sa)		20	56	3.57	0.637	0.622	1.347
(If)		18	59	1.69	0.678	0.667	1.465
(Ni)		20	64	4.69	0.661	0.630	1.473
(Di)		20	56	0.00	0.647	0.607	1.369
(Er)		20	59	5.08	0.658	0.690	1.419
(Gr)		19	49	0.00	0.570	0.531	1.185
(Eh)		20	52	3.85	0.595	0.575	1.241

Abbreviations: AGB, total dry aboveground biomass; CEC_pot_, soil potential cation‐exchange capacity; *F*_v_/*F*_m_, ratio of variable to maximal fluorescence; H_e_, expected heterozygosity, H_o_, observed heterozygosity; I, Shannon's diversity index; K, plant‐available soil potassium content; LA, leaf area; LAI, leaf area index; LDMC, leaf dry matter content; N, soil nitrogen content; N_A_, allelic richness; N_ind_, number of evaluated individuals; N_rep_, number of replicates; P, plant‐available soil phosphor content; P_Ap_, private allelic richness; pH, soil reaction; PI, performance index; RH, releasing height; SLA, specific leaf area; soil potassium content; SPI, stomatal pore area index; SPS, stomatal pore surface.

Per replicate, we assessed GPS coordinates and altitude [m.a.s.l.] (eTrex 30, Garmin GmbH, Garching, Germany), slope exposure [°] and slope [°] (TruPulse 200/B Laser Rangefinder, Laser Technology Inc., Lincoln, USA). In addition, LAI (leaf area index; LAI‐2200 Plant Canopy Analyzer, LI‐COR Inc., Lincoln, USA) and soil depth [cm] were measured five and ten times, respectively. We also took five random soil samples per replicate (2 m × 2 m, 4 m^2^), which were mixed and dried. Potential cation‐exchange capacities [cmol/kg] were determined as a measure of all potentially exchangeable cations in total (CEC_pot_), for sodium (CEC_Na_), potassium (CEC_K_), calcium (CEC_Ca_), and magnesium (CEC_Mg_). Soil reaction was obtained by using soil suspension mixed with deionized water (pH_H2O_, pH) and potassium chloride (pH_KCl_). Furthermore, soil contents were analyzed concerning organic carbon (C_org_) [%], lime (C_anorg_ × 8.3 = CaCO_3_) [%], nitrogen (N) [%], plant‐available phosphor (P) [mg/100 g], and plant‐available potassium (K) [mg/100 g]. All soil analyses were conducted in the soil laboratory of the Thüringer Landesanstalt für Landwirtschaft und Ländlichen Raum (TLLLR) following standardized protocols. Mean annual temperature (T_a_) and mean annual precipitation (P_a_) were interpolated for study locations using ArcMap (vers. 10.5; ESRI Inc., Redlands, USA) and data from WorldClim 1.4 global climate database from 1960 to 1990 (Hijmans, Cameron, Parra, Jones, & Jarvis, 2005; www.worldclim.org).

### 2.5 Population genetics and laboratory work

We isolated DNA of sampled individuals from approximately 20–25 mg dry leaf material using a modified CTAB protocol (Doyle & Doyle, 1987; Saghai‐Maroof, Soliman, Jorgensen, & Allard, 1984; modified by adding 1% PVP to CTAB buffer). Nine microsatellite loci were applied to quantify genetic diversity of *T. montanum* populations (Matter, Määttänen, Kettle, Ghazoul, & Pluess, 2012). We conducted PCRs with labeled primers (IRD 700/IRD 800) in 10 µl reaction volumes containing 1× Dream Taq buffer (Thermo Fisher Scientific Inc., Waltham, USA), 0.2 mM dNTPs, 0.1 µM (IRD 700)/0.2 µM (IRD 800) of tailed forward primer, 0.1 µM (IRD 700)/0.2 µM (IRD 800) reverse primer, 0.025 U * µl^−1^ Dream Taq polymerase (Thermo Fisher Scientific Inc., Waltham, USA), and 1.5 µl of undiluted template DNA. PCR cycling was performed using primer‐specific annealing temperatures (t_a_): 65°C (ats002 and ats032), 51°C (ats006), 58°C (ats029), 56°C (Tm10 and Tm12), 55°C (Tm21 and Tm24), and 61°C (Tm16). We carried out locus‐specific touchdown programs to increase PCR specificity (Korbie & Mattick, 2008). Programs comprised (a) initial denaturation 95°C/15 min; (b) 11× [denaturation 95°C/30 s, primer‐specific t_a_ in touchdown (t_a_ + 5°C) to (t_a_) to (t_a_ − 5°C)/ 45 s, extension 72°C/45 s]; (c) 9× (IRD 700) and 15× (IRD 800) [denaturation 95°C/30 s, primer‐specific t_a_/45 s, extension 72°C/45 s]; 15× (d) [denaturation 95°C/30 s, annealing 53°C/45 s, extension 72°C/45 s]; and (e) final extension 72°C/30 min. All PCRs were stored at 4°C. We combined differently labeled PCR products for electrophoretic analyses of fragment lengths using a LI‐COR Long Readir 4200 (Global Edition IR2 DNA Sequencer, LI‐COR Inc., Lincoln, USA).

### 2.6 Data analyses

All statistical analyses (except genetic diversity calculations) were performed with R vers. 3.6.0 (R Core Team, 2019). We calculated means for numerical variables, medians for the ordinal variable slope exposure, and coefficients of variation (CV) for diversity variables as the ratio of standard deviation to mean. To evaluate the multiple linear regression model, we exceptionally used the adjusted coefficient of determination ($R_{\text{adj}}^{2}$) instead of the coefficient of variation (*R* ^2^). This has the benefit of avoiding model overfitting in *R* ^2^ calculation (see Crawley, 2007).

#### 2.6.1 Intraspecific functional trait variation (iFD_CV_)

To detect erroneous entries (errors in measurement) in functional traits, we excluded all records (seven trait measurements in total) from the dataset with a distance of >4 standard deviations from the mean of all individuals (compare Díaz et al., 2016; Kattge et al., 2011). We deleted F_v_/F_m_ outliers and the respective PI values (identical source, Pocket PEA). We also checked collinearity among traits, that is, when two or more traits were highly correlated (*r* > ~│.7│; Dormann et al., 2013). We assessed correlations among functional traits with Spearman's rank coefficient (r_SP_, cor.test()) due to non‐normal distribution of data. The r package “corrgram” vers. 1.13 (Wright, 2018) was used to visualize correlations. As correlation coefficient values (r_Sp_) were below ~│.7│, we did not exclude particular functional traits (see Figure S1).

ITV (or functional diversity, “FD”) can be calculated in different manners. Functional trait dissimilarity among or within species is used to calculate FD via trait distance matrices and dendrograms (Petchey & Gaston, 2006; Tilman, 2001; see individual‐based FD (iFD) calculations by Cianciaruso, Batalha, Gaston, & Petchey, 2009 across species and Wood, McKinney, & Loftin, 2017 within species). Hypervolumes can also be applied to capture trait space and variation. However, axes (traits) sometimes have to be reduced to follow recommendations about the ratio between observations and number of dimensions and orthogonality among traits (e.g., to four axes, see Benavides, Scherer‐Lorenzen, & Valladares, 2019). The “iFD_CV_” method used herein is a trait‐by‐trait approach simply incorporating individual trait variation based on population‐wise coefficients of variation (CVs) within a species (see, e.g., Helm et al., 2019). We ensured the absence of collinearity among functional traits (see above), and thus, there was no further need to do axes (trait) reductions like in hypervolume approaches. Our concept focuses on the functional variation within the population of a species, instead of functional differences. Each trait contributes independently and with different weight to the index because standardized trait CVs have different value ranges (variation), and, for example, traits with larger variation contribute stronger to iFD_CV_ than traits with a smaller variation. Therefore, our population‐wise trait‐by‐trait approach (iFD_CV_) is appropriate for studying environmental or genetic effects on ITV.

We assessed population‐wise iFD_CV_ as the mean CV of RH (CV_RH_), AGB (CV_AGB_), LA (CV_LA_), SLA (CV_SLA_), LDMC (CV_LDMC_), *F*_v_/*F*_m_ (CV_Fv/Fm_), PI (CV_PI_), SPS (CV_SPS_), and SPI (CV_SPI_; Tables 1 and 2; see also Helm et al., 2019 for CV functional trait calculation).

#### 2.6.2 Within‐habitat heterogeneity (HD)

We tested for correlations among environmental factors with Spearman's rank coefficient (*r*_SP_, cor.test()) due to non‐normal distribution of data. The r package “corrgram” vers. 1.13 (Wright, 2018) was used to visualize correlations. As explained above for functional traits, we checked for collinearity (*r* > ~│.7│; Dormann et al., 2013) and excluded the factors CEC_K_, CEC_Ca_, pH_KCl_, C_org_, CaCO_3_, T_a_, P_a_ (*r*_SP_ > .7), and CEC_Mg_ (*r*_SP_ = .50), and one variable due to an almost complete absence of variation (CEC_Na_; see Figure S2 for correlations). Afterward, we calculated abiotic within‐habitat heterogeneity (HD) as location‐wise mean CV of altitude (CV_altitude_), slope exposure (CV_slope exposure_), slope (CV_slope_), leaf area index (CV_LAI_), soil depth (CV_soil depth_), potential soil cation‐exchange capacity (CV_CECpot_), pH (CV_pH_), soil nitrogen content (CV_N_), soil phosphor content (CV_P_), and soil potassium content (CV_K_; see Tables 1 and 2).

#### 2.6.3 Genetic diversity (GD)

**FIGURE 3:**
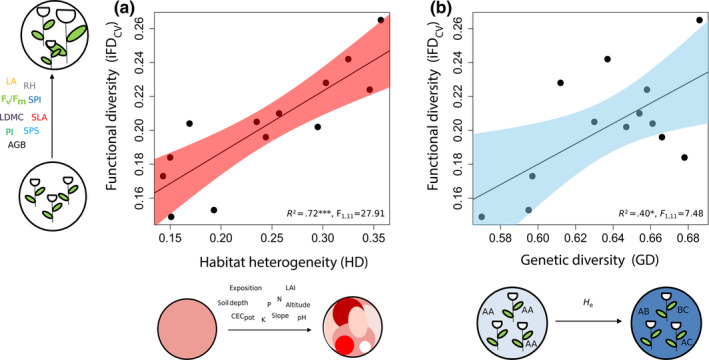
Significant positive relationships between iFD_CV_ and (a) abiotic within‐habitat heterogeneity and (b) genetic diversity (H_e_) including 13 *Trifolium montanum* populations (*n* = 255 to 260 individuals) of Central Europe. Confidence intervals (95%) are drawn. See Table 2 for abbreviations. Significance levels: ***p < .001 and *p < .05

**FIGURE 4:**
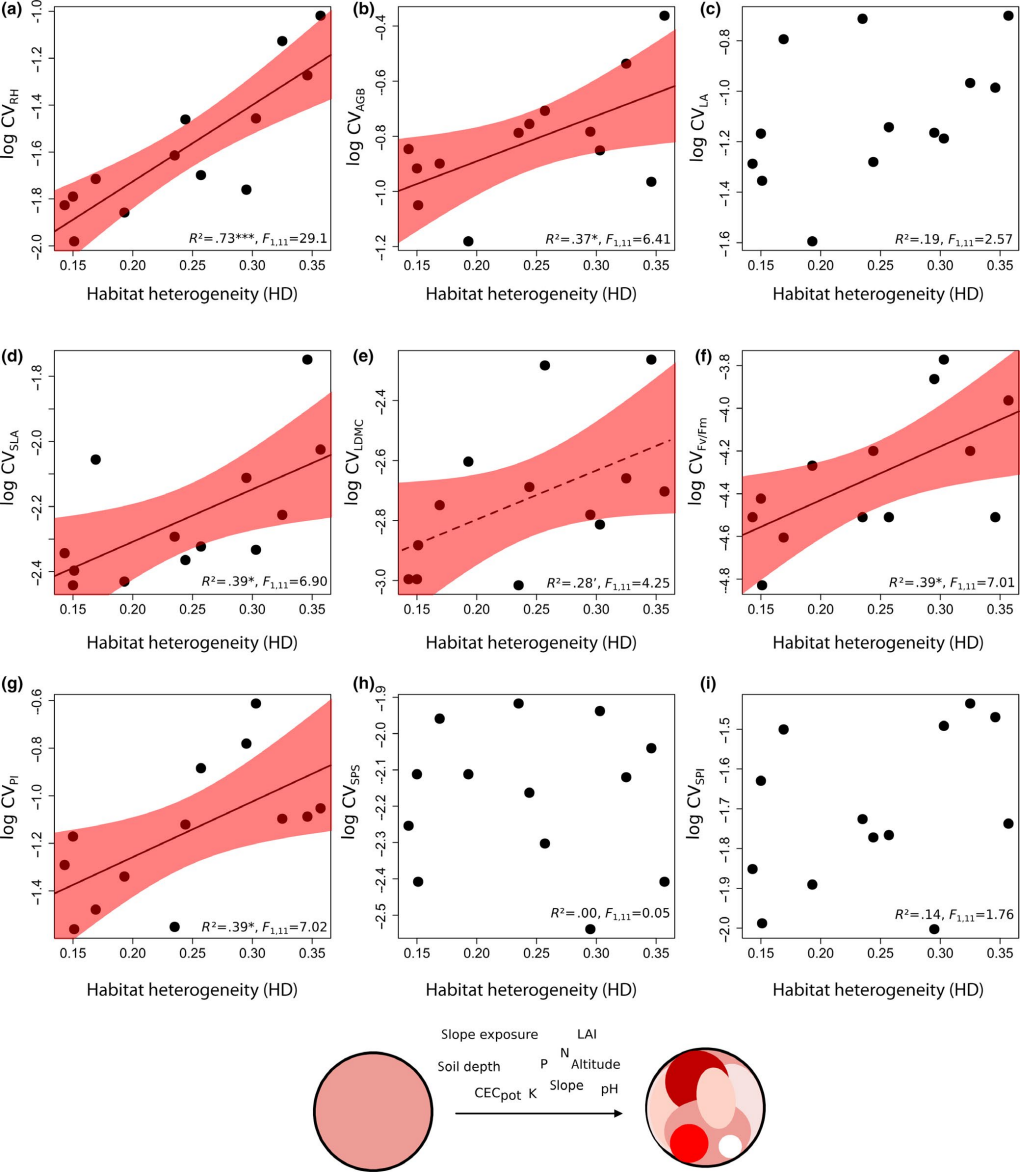
Relationships between coefficient of variation of particular traits (CV_traits_) and abiotic within‐habitat heterogeneity (HD) in 13 *Trifolium montanum* populations (*n* = 260 individuals) of Central Europe. 95% confidence intervals are drawn for all (marginal) significant relationships. Dotted regression lines represent only marginal significant relationships (0.05 < *p* < .1). See Table S1 for detailed model statistics, and Table 2 for abbreviations. Significance levels: ***p < .001, *p < .05 and ‘=.1 > *p *> .05

**FIGURE 5:**
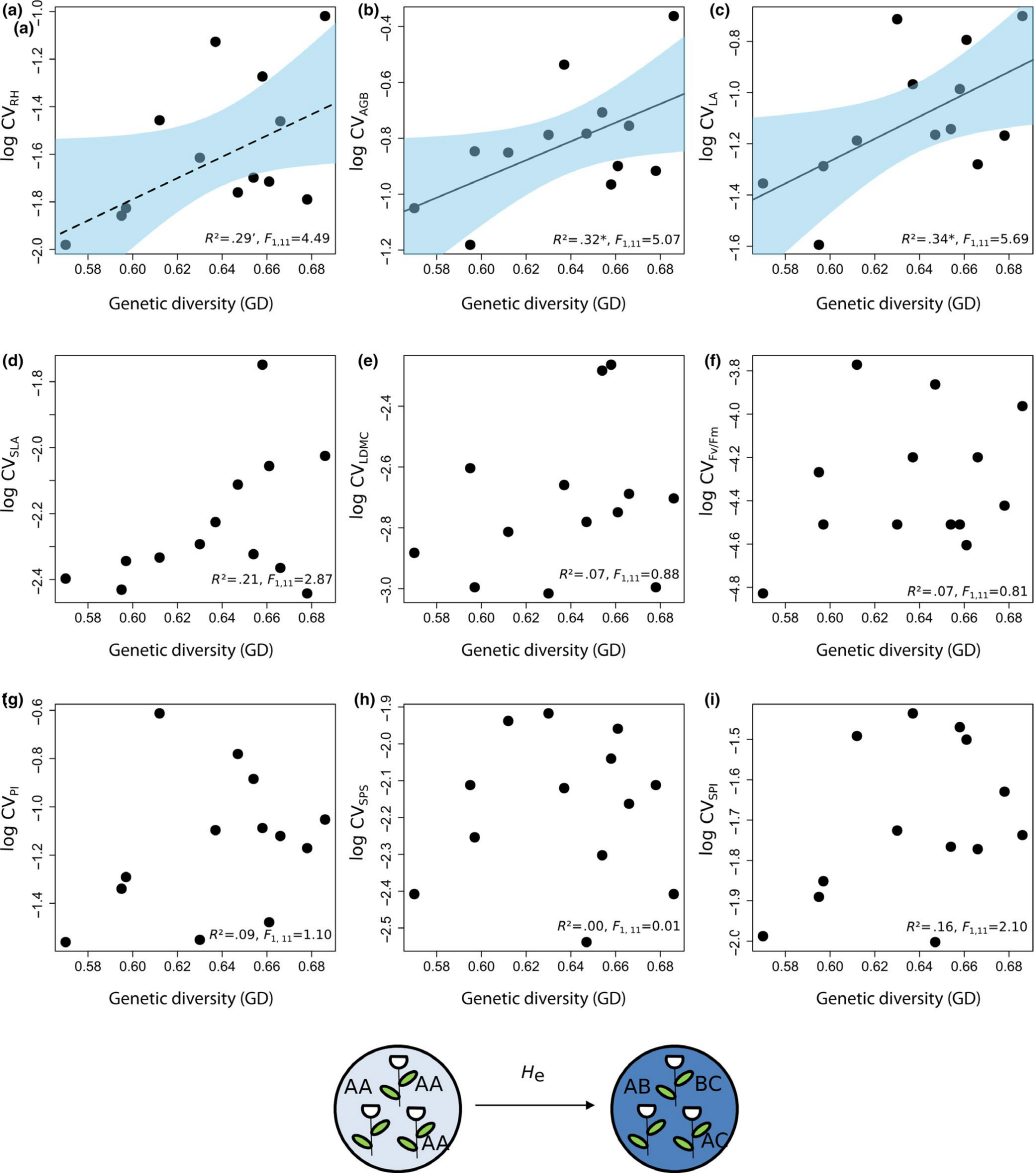
Relationships between coefficient of variation of particular traits (CV_traits_) and genetic diversity (GD, H_e_) in 13 *Trifolium montanum* populations (*n* = 255 individuals) of Central Europe. 95%‐confidence intervals are drawn for all (marginal) significant relationships. Dotted regression lines represent only marginal significant relationships (.05 < *p* < .1). See Table S1 for detailed model statistics, and Table 2 for abbreviations. Significance levels: *p < .05 and ‘=.1 > *p*> .05

We scored microsatellite fragments with an internal size standard. We proved the scoring procedure at least three times and removed ambiguous results. Analyses were conducted for all individuals characterized by at least four microsatellite loci resulting in a final sample size of *n* = 255 individuals (see Table 2). Mean loci coverage was 90% per individual, that is, in mean, 90% of loci were present in an individual. To ensure that sampled populations represent the same genetic line of the species, we calculated individual‐ and population‐wise distance matrices based on Nei´s genetic distance (Nei, 1978) and conducted principal coordinate analyses (PCoAs) in GenAlEx vers. 6.503 (Peakall & Smouse, 2006, 2012). Moreover, we performed analyses in STRUCTURE vers. 2.3.4 (Pritchard, Stephens, & Donnelly, 2000) setting an admixture model (with correlated allele frequencies), burn‐in to 5,000, MCMC to 50,000, and K to one to 13 (10 replicates per K). The optimal K was determined by STRUCTURE HARVESTER (Earl & vonHoldt, 2012) using the Evanno method. We merged the replicates of each optimal *K* (*K* = 2 and *K* = 9) with CLUMPP vers. 1.1.2 (Jakobsson & Rosenberg, 2007) and plotted results with DISTRUCT vers. 1.1 (Rosenberg, 2004). PCoA and STRUCTURE results showed that all populations have high levels of admixture and the geographically distant population KW does not belong to a separate genetic lineage of *T. montanum* (see Figures S3–S5). Moreover, relationships among iFD_CV_, HD, and GD in KW fit those observed among Central German populations (see Table 1 and Figures 3, 4, 5). To examine the effect of genetic diversity on iFD_CV_, we were careful to sample *T. montanum* populations of different sizes (about 50–20,000 individuals, Karbstein et al., unpubl. data). Population size of *T. montanum* was positively related to genetic diversity (Karbstein et al., unpubl. data), and varying genetic diversity is needed to examine its effect on iFD_CV_.

We calculated population‐wise multilocus allelic richness (N_A_) and mean values of private allelic richness (P_Ap_), observed (H_o_) and expected heterozygosity (H_e_), and Shannon´s diversity index (I), using GenAlEx vers. 6.503 (Hardy, 1908; Nei, 1973, 1973, 1978; Peakall & Smouse, 2006, 2012; Shannon, 1948; Weinberg, 1908; see Tables 1 and 2).

#### 2.6.4 Saturation of iFD_CV_, HD and GD

To examine whether iFD_CV_, HD, and GD (see Statistical modeling section) are saturated within populations/habitats, we used an r script (Karbstein, 2020) that randomly chose one to 20, one to five and one to 18 (18 individuals genotyped per population at a minimum) samples and calculated iFD_CV_, HD and GD, respectively with 100 iterations per step. Mean iFD_CV_, HD and GD values were plotted against sample size for each population/habitat. We used r functions implemented in the packages “dbplyr” vers. 1.4.2 (Wickham & Ruiz, 2019) to randomly sample populations/habitats and “adegenet” vers. 2.1.1 (Jombart et al., 2018) to calculate expected heterozygosity (H_e_).

#### 2.6.5 Statistical modeling

To assess whether iFD_CV_ is related to within‐habitat heterogeneity and genetic diversity (and to assess which specific genetic diversity index best explains iFD_CV_), we employed a multiple linear regression model with iFD_CV_ as the dependent variable, and HD and genetic diversity indices N_A_, P_Ap_, H_o_, H_e,_ and I as explaining, independent variables. To fulfill statistical assumptions, we standardized independent variables to zero mean for unit variance (z‐transformation). We conducted linear regression model simplification with the standard backward selection approach by always excluding the least significant variable (*p* > .1) until the final minimal adequate model was attained (see Crawley, 2015). Next, we carried out an ANOVA implemented in the R function aov() and additionally calculated the Akaike information criterion (AIC) with AIC() for model comparison to justify each simplification step. We checked our final model using the R function plot(), and it visually fulfilled the assumptions for normality, homoscedasticity, and linearity. The final multiple linear regression model contained HD and expected heterozygosity (H_e_) as explaining, independent variables (see Table S1). H_e_ is widely used as genetic diversity index, and it less depends on population history (e.g., bottlenecks) compared to the other indices (Freeland et al., 2011; Kalinowski, 2004; Szczecińska, Sramko, Wołosz, & Sawicki, 2016). Therefore, we used population‐wise multilocus mean values of expected heterozygosity (H_e_) as genetic diversity (GD; see also section Discussion, Relationships between intraspecific functional trait variation and genetic diversity).

To illustrate separate relationships between iFD_CV_ and HD or GD (see Figure 3), we executed two linear regression models and plotted regression results. Additionally, we conducted a linear regression model to assess whether HD is associated with GD. GD was handled as dependent and HD as the independent variable.

To assess which functional traits were related to HD and/or GD (see Table S1), we performed linear regression models with log‐transformed trait variation as dependent variable and HD or GD as independent variable. We log‐transformed the dependent model variable to achieve normality and/or linearity, and checked normality with the Shapiro–Wilcox test. Model assumptions were again visually examined as described above.

To test for spatial autocorrelation among populations/habitats (e.g., closer populations with more similar genetic diversity and more similar iFD_CV_) within linear regression models, we calculated Moran's I (Moran, 1950) values using the R function correlog() function included in the r package “ncf” vers. 1.2‐6 (Bjornstad & Cai, 2018) based on the model residuals. Moran's I values mostly resided around ≤±1. Permutation of two‐sided *p* values per distance class (with 1,000 resamples under the null distribution) comprised only six to 11% significant *p* values on average with different increment settings across linear regression models indicating only very weak spatial autocorrelation. Hence, we did not consider models accounting for spatial autocorrelation.

To support the interpretation of results found between iFD_CV_ and HD, we also examined correlations among particular functional traits and abiotic environmental factors chosen to calculate HD. We added a value of 1 to all CVs and logarithmized traits and environmental factor CVs (CVs have to be >1) to achieve normal distribution. We used the rcorr() function within the r package “hmisc” vers. 4.2‐0 (Harrell, 2019) to calculate a correlation matrix based on Pearson's rank correlation coefficient. We carried out the corrplot() function implemented in the r package “corrplot” vers. 0.84 (Taiyun & Simko, 2017) to visualize the correlation matrix only considering *p* values below .1.

## 3 RESULTS

### 3.1 Relationships among iFD_CV_, HD, and GD

The results of the multiple linear regression model demonstrated that iFD_CV_ was positively and significantly related to abiotic within‐habitat heterogeneity (HD) and genetic diversity (H_e_, GD), accounting for 67.42% (*p* < .001) and 32.58% (*p* < .05) of explained variation, respectively (see Table S1). Together both aspects significantly explained 77% of iFD_CV_ ($R_{\text{adj}}^{2}$ = .77, *F*_2, 10_ = 21.66, *p* < .001).

Individual relationships between iFD_CV_ and HD, and iFD_CV_ and GD were also statistically significant (Figure 3): The iFD_CV_ formed a well‐explained positive linear relationship with HD (*R* ^2^ = .72, *F*_1, 11_ = 27.91, *p* < .001) whereas a linear, though weaker, relationship was found between iFD_CV_ and GD (*R* ^2^ = .40, *F*_1, 11_ = 7.48, *p* < .05).

HD and GD of *T. montanum* populations were not significantly related in our study (*R* ^2^ = .18, *F*_1, 11_ = 2.37, *p* = .15). We observed saturation of all diversity variables. For iFD_CV_, HD and GD, the curves began to saturate between 5–10, 4–5, and 10–15 samples, respectively (see Figures S6–S8).

### 3.2 Relationships of functional traits with environmental factors and genetic diversity

Most functional traits were significantly positively related to HD and some to GD (Figures 4 and 5; see Table S1 for detailed statistics). We observed a higher number of (marginally) significant relationships between functional traits (CV_RH_, CV_AGB_, CV_SLA_, CV_LDMC_, CV_Fv/Fm,_ and CV_PI_) and HD than between functional traits (CV_RH_, CV_AGB_ and CV_LA_) and GD. CV_RH_ is strongest related to HD (*R* ^2^ = .73), followed by CV_SLA_ (*R* ^2^ = .39), CV_Fv/Fm_ (*R* ^2^ = .39), CV_PI_ (*R* ^2^ = .39), CV_AGB_ (*R* ^2^ = .37), and CV_LDMC_ (*R* ^2^ = .28). With GD, CV_LA_ (*R* ^2^ = .34), CV_AGB_ (*R* ^2^ = .32), and CV_RH_ (*R* ^2^ = .29) exhibited significant relationships of similar strength.

**FIGURE 6:**
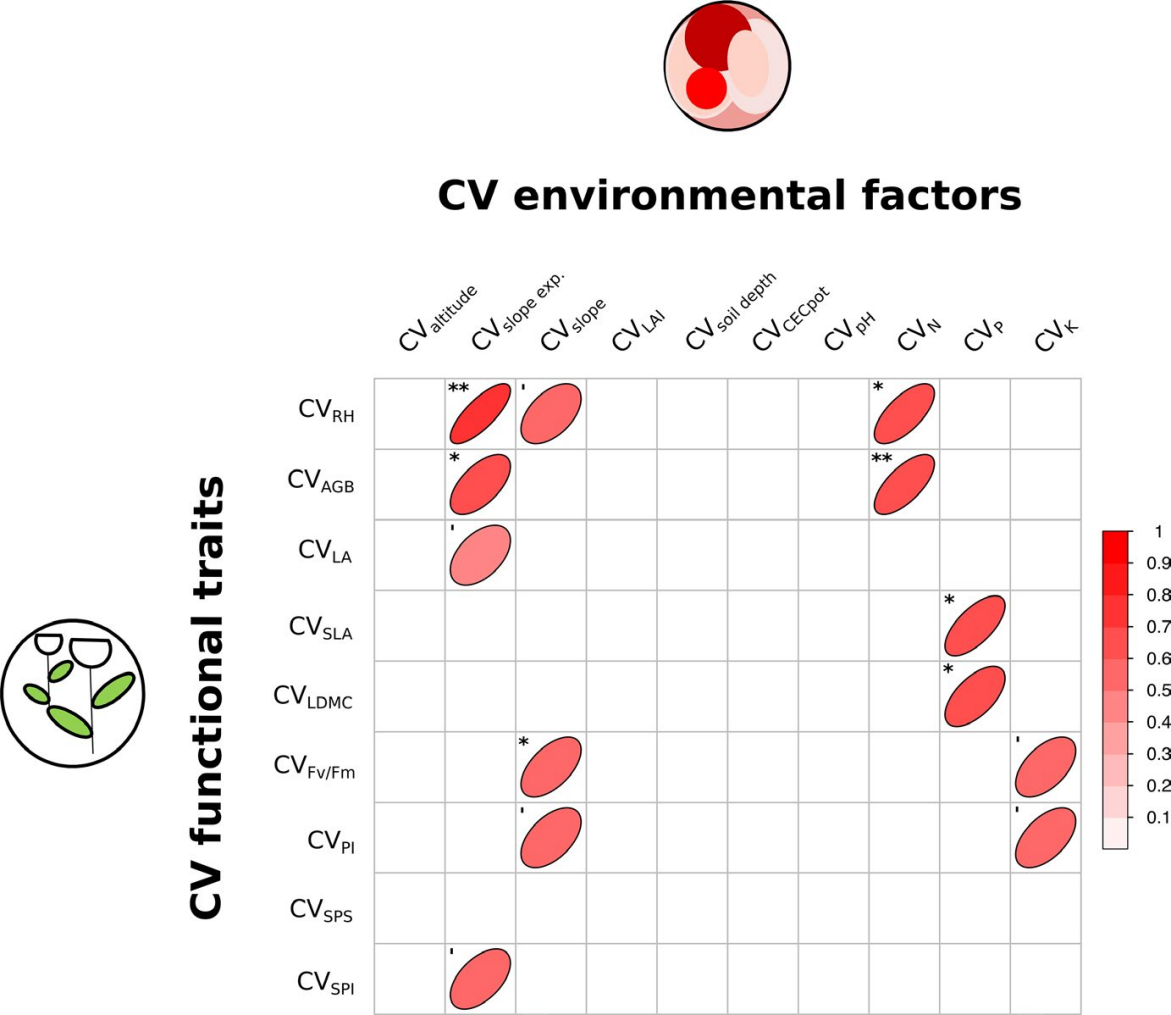
Visualized correlation matrix based on Pearson correlation coefficients between variation of particular traits (CV_trait_) and particular abiotic environmental factors (CV_factor_) in 13 *Trifolium montanum* populations (*n* = 260 individuals) of Central Europe. We only illustrated (marginal) significant results (see Results). Width of an ellipse reflects the correlation coefficient, that is, the higher a correlation coefficient (in positive and negative direction), the narrower the ellipse. See Table 2 for abbreviations, and Table S2 for statistics. Significance levels: **=*p* < .01, *=*p* < .05 and ‘=0.1 > *p *> .05

We found (marginally) significant positive correlations between functional traits and abiotic environmental factors chosen to calculate HD (Figure 6, see Table S2): Some trait CVs are positively correlated to CV_slope exposure_ (CV_RH_, CV_AGB_, CV_LA,_ and CV_SPI_) and CV_slope_ (CV_RH_, CV_Fv/Fm_ and CV_PI_). Many traits were positively correlated to CV of soil nutrients, that is, CV_N_ (CV_RH_ and CV_AGB_), CV_P_ (CV_SLA_ and CV_LDMC_), and CV_K_ (CV_Fv/Fm_ and CV_PI_). No significant trait CV correlations were found to CV_altitude_, CV_LAI_, CV_soil depth_, CV_CECpot,_ and CV_pH._ Correlations between trait CVs and environmental CVs revealed widely positive coefficients (~71%).

## 4 DISCUSSION

Connecting intraspecific functional trait variation with genetic and environmental variation is an important ecological challenge. This study showed that population‐wise intraspecific functional trait variation (iFD_CV_) of *T. montanum* can be attributed to a high extent (77%) to both abiotic within‐habitat heterogeneity and population genetic diversity under natural environmental conditions (Figure 3). Interestingly, within‐habitat heterogeneity statistically affected iFD_CV_ considerably stronger than genetic diversity (Figures 4 and 5).

### 4.1 Relationships between intraspecific functional trait variation and within‐habitat heterogeneity

Variation of morphology‐related functional traits RH, AGB, and LA was mainly correlated to variation of habitat slope exposure and slope, whereas variation of (eco‐)physiology‐related traits (SLA, LDMC, F_v_/F_m_, PI, SPS, and PCI) was predominantly correlated to variation of slope characteristics and soil factors (Figure 6). Within a habitat, different slopes and slope exposures, influencing soil humidity, may have led to an increase or reduction in height, biomass, and leaf area of *T. montanum*. For example, drought stress is known to limit nutrient uptake and thus photosynthesis and plant growth (Farooq, Wahid, Fujita, & Basra, 2009; Jaleel et al., 2009; Pérez‐Harguindeguy et al., 2013). Also, RH and AGB were positively associated with soil nitrogen content (N), and N‐deficient soils in *T. montanum* habitats probably constrained height and biomass accumulation of individuals as well (see, e.g., Ågren, Wetterstedt, & Billberger, 2012). (Positive) associations between plant height or biomass to soil properties and particularly N are in line with literature (Cornelissen et al., 2003; Razaq, Zhang, & Shen, 2017; Reich & Hobbie, 2013). In contrast, variation of SLA and LDMC was correlated to soil phosphorous content (P). Phosphor regulates protein biosynthesis and development of new plant tissue (see Kerkhoff, Fagan, Elser, & Enquist, 2006), and P‐deficient soils may thus impact relative growth rate and leaf robustness. SLA and LDMC respond also to soil properties (Cornelissen et al., 2003). P has been shown to form only rare and weak positive intraspecific relationships with SLA and particularly in herbaceous species when N is in abundant supply (wild rice; Dwyer, Hobbs, & Mayfield, 2014; Sims, Pastor, & Dewey, 2012). Moreover, soil potassium content (K), besides slope affecting soil humidity (see above), influenced performance and vitality (PI and *F*_v_/*F*_m_). High soil potassium content was mainly found in CaCO_3_‐rich soils, which *T. montanum* prefers (Jäger, 2011). Due to physiological constraints, low K and CaCO_3_ conditions may decrease the performance/vitality of *T. montanum* individuals while high K and CaCO_3_ may increase it. Studies already revealed that *F*_v_/*F*_m_ and PI respond positively to increased calcium supply due to stabilization of chlorophyll and an increase of photosystem II activity (*Coffea arabica*; Ramalho, Rebelo, Santos, Antunes, & Nunes, 1995; *Zea mays* and *Solanum lycopersicum* cultivars; Kalaji et al., 2014). In general, several functional traits may respond simultaneously to environmental heterogeneity in *T. montanum* habitats. Intraspecific functional trait variation captured by iFD_CV_ is probably the response of populations to increased abiotic and biotic environmental differences within their habitats (see also Ghalambor et al., 2007; Nicotra et al., 2010, 2015): as shown, iFD_CV_ was positively correlated to within‐habitat heterogeneity, suggesting that the more environmentally variable a habitat, the higher the intraspecific functional trait variation in *T. montanum* populations. Results are line with literature showing associations between functional trait values and diversity, and environmental conditions within and across species (e.g., Albert et al., 2010; Bernhardt‐Römermann, Gray, et al., 2011; Bucher et al., 2016; Díaz & Cabido, 2001; Gratani, 2014; Karbstein et al., 2019; König et al., 2018; Violle et al., 2007). Intraspecific functional differences are known to facilitate a more flexible response to varying abiotic conditions (see, e.g., Bucher et al., 2016 for elevational gradients; Karbstein et al., 2019 for small‐scale habitat differences). Thus, iFD_CV_ likely affects population growth and reproduction with positive consequences for survival and fitness (see also Nock et al., 2016; Violle et al., 2007).

### 4.2 Relationships between intraspecific functional trait variation and genetic diversity

Genetic diversity, in terms of microsatellite variation, was positively related to intraspecific functional trait variation (iFD_CV_; Figures 3b and 5). This observation is also in line with literature indicating positive relationships between trait variation/plant fitness (iFD_CV_, plant fitness, see above) and genetic diversity within species (e.g., Leimu et al., 2006; Waitt & Levin, 1998). Microsatellite markers are frequently applied to capture population genetic diversity (e.g., Matter et al., 2013; Matter et al., 2012; Prinz, Weising, & Hensen, 2009). They are widely distributed throughout genomes, and while regulatory functions in gene expression are known, these markers are presumed to predominantly occur in non‐coding regions and thus to be under neutral selection (see also Ellegren, 2004; Li, Korol, Fahima, Beiles, & Nevo, 2002; Vieira, Santini, Diniz, & Munhoz, 2016). The weak relationship of iFD_CV_ with genetic diversity may be explained by the neutrality of applied microsatellite markers in relation to selected functional traits. However, our intention was not to explain functional traits with particular microsatellites but to assess whether iFD_CV_ and/or particular trait variation coincide with genetic diversity.

It is likely that genetic diversity limits and influences the range of iFD_CV_. Mutation and recombination events create genetic variation and thus novel functional trait variation within a population. Natural selection probably acts on the genetic basis of trait variation, which in turn probably affects the range of iFD_CV_. After modeling, expected heterozygosity (H_e_) best‐explained iFD_CV_. H_e_ is based on allelic structure, represents genotype and allele frequencies, and is less sensitive to population history (Freeland et al., 2011; Kalinowski, 2004; Szczecińska et al., 2016). Heterozygosity within individuals and populations probably influenced iFD_CV_ because it enhances the reaction norm and adaptability and thus affects intraspecific trait variation (Boulding, 2008; Freeland et al., 2011; Reed & Frankham, 2003). Interestingly, only the variation of morphology‐related traits was associated with genetic diversity in *T. montanum* (see also Waitt & Levin, 1998). In contrast, both variation of morphology‐ and (eco‐)physiology‐related traits was linked to within‐habitat heterogeneity. (Eco‐)physiology‐related traits (gas exchange and photosynthesis)  tend to have a  higher heritability (trait variation due to genetic variation) compared to morphology‐related traits (morphology and vegetative performance, Geber & Griffen, 2003), and should thus be more sensitive to genetic variation . However, an explanation might be that heterozygosity effects (see, e.g., Boulding, 2008; Freeland et al., 2011; Reed & Frankham, 2003) are stronger pronounced in morphology‐related traits leading to similar trait variation and genetic variation based on microsatellites.

However, a positive relationship between genetic diversity and iFD_CV_ may be strengthened by the self‐incompatible nature of *T. montanum* (see, e.g., Leimu et al., 2006; Reed & Frankham, 2003; Schleuning et al., 2009). Observations between classical fitness parameters and genetic diversity of self‐incompatible species are frequent and can be explained by pollinator limitation in fragmented populations with a low density of flowering individuals (Leimu et al., 2006; Schleuning et al., 2009). This process enhances the loss of genetic diversity in smaller populations, and it extends the range of genetic variation between small and big populations, probably also affecting the range of IFD_CV_. Thus, relationships between genetic diversity and iFD_CV_, which influences plant fitness directly and indirectly (Nock et al., 2016; Violle et al., 2007), might be strengthened in *T. montanum*.

### 4.3 Differentiated view on relationships among intraspecific functional trait variation, within‐habitat heterogeneity, and genetic diversity

**FIGURE 7:**
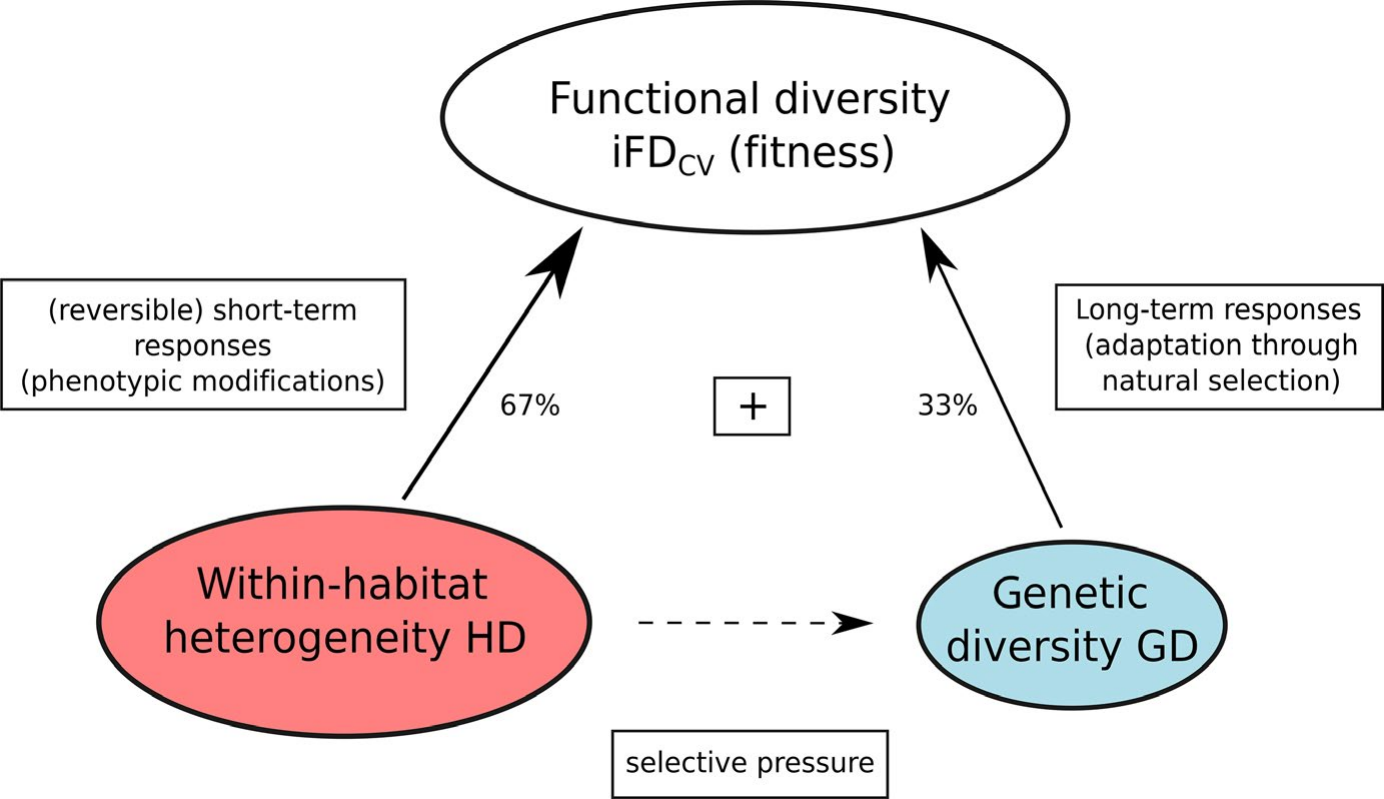
A conceptual model of relationships among intraspecific trait variation (functional diversity; iFD_CV_), abiotic within‐habitat heterogeneity (HD), and genetic diversity (GD) in *T. montanum*. HD influenced iFD_CV_ twice as much as GD symbolized by different circle sizes and arrow strengths. Results and percents are extracted from the multiple linear regression model ($R_{\text{adj}}^{2}$ = .77, *F*_2, 10_ = 21.66, *p* < .001). HD can lead to (reversible) short‐term responses, that is, to modification of functional trait expression (phenotypic modifications, variation). In contrast, GD is controlled by selection and is a prerequisite for adaptation through natural selection. iFD_CV_ thus also depends on the available genetic variation within a population. Habitat heterogeneity and genetic diversity are not significantly related in this study (*R* = .10, *F*_1, 11_ = 2.37, *p* = .15; dashed line)

Relations between iFD_CV_, within‐habitat heterogeneity and genetic diversity (Figure 7) are reported from several species and discussed in literature cited above. Under natural environmental conditions, within‐habitat heterogeneity and genetic diversity probably act on iFD_CV_ in complex ways. For example, several factors may influence whether environmental variation within a habitat promotes the occurrence of different genotypes. Although a positive relationship between within‐habitat heterogeneity and population genetic diversity was expected from literature (particularly for self‐incompatible, outcrossing species, like *T. montanum*, with moderate to high population genetic diversity), we did not observe a significant effect. Several reasons can explain this result. Large phenotypic plasticity of traits can enable individuals to inhabit different environmental niches potentially shielding them from natural selection (e.g., Ghalambor et al., 2007). Thus, there would have been no need to select for higher functional trait variation, weakening the relationship between within‐habitat heterogeneity and population genetic diversity. Moreover, some close *T. montanum* populations are potentially connected (or were at least in the past) due to sheep and goat grazing (transhumance). Connectivity would have allowed for gene flow among them (Linhardt & Grant, 1996; Reisch & Schmid, 2019; Vellend & Geber, 2005) superseding (partially) local genotypes and altering population genetic diversity and adaptation to local environmental habitat conditions. However, some *T. montanum* populations in lowly/highly variable habitats are characterized by low/high genetic diversity (and low/high iFD_CV_) indicating an association between environment and genetics (and trait variation) within habitats of a species (see also Gram & Sork, 2001; Huenneke, 1991; Linhardt & Grant, 1996). Varying resource exploitation of different genotypes may explain the observed pattern (see, e.g., Agashe & Bolnick, 2010; Reusch et al., 2005). Moreover, within‐habitat heterogeneity could be underestimated in large *T. montanum* habitats (e.g., Er, If, Bo, St) characterized by large population sizes (Karbstein et al., in prep.), genetic diversity, and iFD_CV_. More environmental replicates would have potentially led to higher within‐habitat heterogeneity estimates additionally strengthening positive relationships among iFD_CV,_ within‐habitat heterogeneity, and genetic diversity.

Saturation of diversity variables (iFD_CV_, HD, and GD) is an important feature. Unsaturated variables can bias relationships, and can lead to false results and conclusions. In *T. montanum*, at least five to 10 samples were sufficient to saturate iFD_CV_ within populations (see also Bastias et al., 2017). Within‐habitat heterogeneity was saturated by three to five samples per habitat, and genetic diversity reached the plateau at 10 to 15 samples per population, not biasing our regression models.

In addition, epigenetic processes, like DNA methylation or activation of transposable elements, in response to environmental variation can also alter phenotypic plasticity (Nicotra et al., 2015; Weinhold, 2006) and thus ITV potentially explaining a particular amount of unexplained variation in regression models. Our primary goal was not to separate the environmental component from the genetic one but to understand the relative importance of both environment and genetics on iFD_CV_, and particularly, how populations react under natural environmental conditions. To clearly separate the environment from the genetic impact on iFD_CV_, common garden experiments under controlled environmental conditions are necessary. Moreover, genomic data will provide more insights into genetic variation of populations, and in investigating relationships between functional trait variation and genetic variation. Connecting intraspecific functional trait, environmental, and genetic variation remains still an important ecological challenge.

### 4.4 Impact on biodiversity research

Trait variation is probably of major importance to plants short‐term adjustment on (rapid) environmental changes (see also Arnold et al., 2019; Gratani, 2014). Genetic diversity influences the range of trait plasticity and thus trait variation within a population, which can be advantageous for short‐term responses (e.g., land use abandonment) by offering genetic variants that are fitter under novel environmental conditions. Moreover, in the long term (e.g., considering anthropogenic climate change), genetic diversity offers variation for natural selection to act and thus allows for adaptation to novel habitat conditions.

Environmental habitat aspects and population genetics should be considered in biodiversity research dealing with intraspecific functional trait variation at population, community, and ecosystem level. Consideration of these aspects can prevent bias and misinterpretation of trait variation analyses, for example, comparing trait variation between sites where trait differences cannot be attributed to environment or genetics. Habitat features are directly extractable from field measurements as shown in this study or potentially from databases with a high spatial resolution (e.g., WorldClim; Hijmans et al., 2005). If genetic features of a species cannot be investigated due to a lack of suitable markers, population size can be used as a cautious proxy of genetic diversity (see Leimu et al., 2006).

Our study demonstrates the potential of deriving intraspecific functional trait variation based on environmental and genetic aspects (or its proxies) and provides empirical evidence to encourage the incorporation of intraspecific functional trait variation into interspecific comparisons (see also Albert et al., 2010; de Bello et al., 2011; Violle et al., 2012). Directly measured species‐specific intraspecific functional trait variation, but also values from databases provide the possibility for a better understanding of community and ecosystem responses to environmental changes and a more realistic estimation of ecosystem functioning.

## CONFLICT OF INTEREST

None.

## AUTHORS' CONTRIBUTION

**Kevin Karbstein:** Conceptualization (supporting); Investigation (equal); Methodology (equal); Software (equal); Visualization (equal); Writing‐original draft (equal); Writing‐review & editing (equal). **Kathleen Prinz:** Conceptualization (lead); Methodology (equal); Project administration (equal); Supervision (equal); Writing‐original draft (equal); Writing‐review & editing (equal). **Frank Hellwig:** Conceptualization (lead); Project administration (equal); Writing‐original draft (supporting); Writing‐review & editing (supporting). **Christine Römermann:** Conceptualization (lead); Formal analysis (equal); Investigation (equal); Methodology (equal); Project administration (equal); Supervision (equal); Writing‐original draft (equal); Writing‐review & editing (equal). 

The study was designed by K.P., F.H., and C.R. K.K. sampled and analyzed the data Statistical data analyses were supported by K.P. and C.R. K.K. wrote the manuscript with equal contributions from K.P., F.H., and C.R.

## Supporting information

Supplementary Material

## Data Availability

Code availability. R scripts used in analyses are available from the corresponding author on request. The specific R script to calculate diversity saturation of iFD_CV_, HD, and GD is available on Github (https://github.com/KK260/saturation‐of‐diversity‐variables). Data availability. Basic data supporting the findings of this study are available within the manuscript. Functional trait, environmental, and genetic data will be made available upon publication on Dryad data repository (https://doi.org/10.5061/dryad.n02v6wwtd). Functional trait data will additionally be deposited on TRY database (www.try‐db.org) upon publication.

## References

[ece36255-bib-0001] Ackerly, D. D. , & Donoghue, M. J. (1998). Leaf size, sapling allometry, and Corner’s rules: Phylogeny and correlated evolution in maples (Acer). The American Naturalist, 152(6), 767–791. 10.1086/286208 18811427

[ece36255-bib-0002] Ackerly, D. D. , Dudley, S. A. , Sultan, S. E. , Schmitt, H. , Coleman, J. S. , Linder, C. R. , … Lechowicz, M. J. (2000). The evolution of plant ecophysiological traits: Recent advances and future directions. BioScience, 50(11), 979–995. 10.1641/0006-3568(2000)050[0979:TEOPET]2.0.CO;2

[ece36255-bib-0003] Agashe, D. , & Bolnick, D. I. (2010). Intraspecific genetic variation and competition interact to influence niche expansion. Proceedings of the Royal Society B: Biological Sciences, 277(1696), 2915–2924. 10.1098/rspb.2010.0232 PMC2982016 20462902

[ece36255-bib-0004] Agrawal, A. A. (2001). Phenotypic plasticity in the interactions and evolution of species. Science, 294(5541), 321–326. 10.1126/science.1060701 11598291

[ece36255-bib-0005] Ågren, G. I. , Wetterstedt, J. Å. M. , & Billberger, M. F. K. (2012). Nutrient limitation on terrestrial plant growth ‐ modeling the interaction between nitrogen and phosphorus. New Phytologist, 194(4), 953–960. 10.1111/j.1469-8137.2012.04116.x 22458659

[ece36255-bib-0006] Albert, C. H. , Thuiller, W. , Yoccoz, N. G. , Douzet, R. , Aubert, S. , & Lavorel, S. (2010). A multi‐trait approach reveals the structure and the relative importance of intra‐ vs. interspecific variability in plant traits. Functional Ecology, 24(6), 1192–1201. 10.1111/j.1365-2435.2010.01727.x

[ece36255-bib-0007] Arnold, P. A. , Kruuk, L. E. B. , & Nicotra, A. B. (2019). How to analyse plant phenotypic plasticity in response to a changing climate. New Phytologist, 222(3), 1235–1241. 10.1111/nph.15656 30632169

[ece36255-bib-0008] Balasooriya, B. L. W. K. , Samson, R. , Mbikwa, F. , Vitharana, U. W. A. , Boeckx, P. , & Van Meirvenne, M. (2009). Biomonitoring of urban habitat quality by anatomical and chemical leaf characteristics. Environmental and Experimental Botany, 65(2–3), 386–394. 10.1016/j.envexpbot.2008.11.009

[ece36255-bib-0009] Bartelink, H. (1997). Allometric relationships for biomass and leaf area of beech (*Fagus sylvatica* L.). Annales Des Sciences Forestieres, 54(31), 39–50. 10.1051/forest:19970104

[ece36255-bib-0010] Bastias, C. C. , Fortunel, C. , Valladares, F. , Baraloto, C. , Benavides, R. , Cornwell, W. , … Kraft, N. J. B. (2017). Intraspecific leaf trait variability along a boreal‐to‐tropical community diversity gradient. PLoS ONE, 12(2), 1–16. 10.1371/journal.pone.0172495 PMC5328268 28241033

[ece36255-bib-0011] Benavides, R. , Scherer‐Lorenzen, M. , & Valladares, F. (2019). The functional trait space of tree species is influenced by the species richness of the canopy and the type of forest. Oikos, 128, 1435–1445. 10.1111/oik.06348

[ece36255-bib-0012] Bernhardt‐Römermann, M. , Gray, A. , Vanbergen, A. J. , Bergès, L. , Bohner, A. , Brooker, R. W. , … Stadler, J. (2011). Functional traits and local environment predict vegetation responses to disturbance: A pan‐European multi‐site experiment. Journal of Ecology, 99(3), 777–787. 10.1111/j.1365-2745.2011.01794.x

[ece36255-bib-0014] Bjornstad, O. N. , & Cai, J. (2018). ncf: spatial covariance functions.

[ece36255-bib-0015] Boulding, E. G. (2008). Genetic diversity, adaptive potential, and population viability in changing environments In Carroll S. P., & Fox C. W. (Eds.), Conservation biology: Evolution in action (pp. 201–222). Oxford, UK: Oxford University Press.

[ece36255-bib-0016] Bucher, S. F. , Auerswald, K. , Grün‐Wenzel, C. , Higgins, S. I. , Garcia Jorge, J. , & Römermann, C. (2017). Stomatal traits relate to habitat preferences of herbaceous species in a temperate climate. Flora: Morphology, Distribution, Functional Ecology of Plants, 229, 107–115. 10.1016/j.flora.2017.02.011

[ece36255-bib-0017] Bucher, S. F. , Auerswald, K. , Tautenhahn, S. , Geiger, A. , Otto, J. , Müller, A. , & Römermann, C. (2016). Inter‐ and intraspecific variation in stomatal pore area index along elevational gradients and its relation to leaf functional traits. Plant Ecology, 217(3), 229–240. 10.1007/s11258-016-0564-2

[ece36255-bib-0018] Bucher, S. F. , Bernhardt‐Römermann, M. , & Römermann, C. (2018). Chlorophyll fluorescence and gas exchange measurements in field research: An ecological case study. Photosynthetica, 56(4), 1161–1170. 10.1007/s11099-018-0809-5

[ece36255-bib-0019] Bucher, S. F. , Feiler, R. , Buchner, O. , Neuner, G. , Rosbakh, S. , Leiterer, M. , & Römermann, C. (2019). Temporal and spatial trade‐offs between resistance and performance traits in herbaceous plant species. Environmental and Experimental Botany, 157, 187–196. 10.1016/j.envexpbot.2018.10.015

[ece36255-bib-0021] Butler, W. L. , & Kitajima, M. (1975). Fluorescence quenching in photosystem II of chloroplasts. BBA ‐ Bioenergetics, 376(1), 116–125. 10.1016/0005-2728(75)90210-8 1125216

[ece36255-bib-0022] Chen, X. , Visser, E. J. W. , de Kroon, H. , Pierik, R. , Voesenek, L. A. C. J. , & Huber, H. (2011). Fitness consequences of natural variation in flooding‐induced shoot elongation in *Rumex palustris* . New Phytologist, 190(2), 409–420. 10.1111/j.1469-8137.2010.03639.x 21261627

[ece36255-bib-0023] Chevin, L. M. , & Hoffmann, A. A. (2017). Evolution of phenotypic plasticity in extreme environments. Philosophical Transactions of the Royal Society B: Biological Sciences, 372(1723), 10.1098/rstb.2016.0138 PMC5434089 28483868

[ece36255-bib-0024] Cianciaruso, M. V. , Batalha, M. A. , Gaston, K. J. , & Petchey, O. L. (2009). Including intraspecific variability in functional diversity. Ecology, 90(1), 81–89. 10.1890/07-1864.1 19294915

[ece36255-bib-0025] Clark, A. J. , Landolt, W. , Bucher, J. B. , & Strasser, R. J. (2000). Beech (*Fagus sylvatica*) response to ozone exposure assessed with a chlorophyll a fluorescence performance index. Environmental Pollution, 109(3), 501–507. 10.1016/S0269-7491(00)00053-1 15092883

[ece36255-bib-0026] R Core Team . (2019). R: A language and environment for statistical computing. Vienna, Austria: Foundation for Statistical Computing Retrieved from http://www.r‐project.org

[ece36255-bib-0027] Cornelissen, J. H. C. , Lavorel, S. , Garnier, E. , Díaz, S. , Buchmann, N. , Gurvich, D. E. , … Poorter, H. (2003). A handbook of protocols for standardised and easy measurement of plant functional traits worldwide. Australian Journal of Botany, 51(4), 335 10.1071/BT02124

[ece36255-bib-0028] Crawley, M. J. (2007). The R book. Chichester, UK: John Wiley & Sons, Ltd 10.1002/9780470515075

[ece36255-bib-0029] Crawley, M. J. (2015). Statistics: An introduction using R (2nd edition). Chichester, UK: John Wiley & Sons.

[ece36255-bib-0030] Csilléry, K. , Ovaskainen, O. , Sperisen, C. , Buchmann, N. , Widmer, A. , & Gugerli, F. (2020). Adaptation to local climate in multi‐trait space: Evidence from silver fir (*Abies alba* Mill.) populations across a heterogeneous environment. Heredity, 124(1), 77–92. 10.1038/s41437-019-0240-0 31182819 PMC6906498

[ece36255-bib-0031] de Bello, F. , Lavorel, S. , Albert, C. H. , Thuiller, W. , Grigulis, K. , Dolezal, J. , … Lepš, J. (2011). Quantifying the relevance of intraspecific trait variability for functional diversity. Methods in Ecology and Evolution, 2(2), 163–174. 10.1111/j.2041-210X.2010.00071.x

[ece36255-bib-0032] Díaz, S. , & Cabido, M. (2001). Vive la différence: Plant functional diversity matters to ecosystem processes. Trends in Ecology & Evolution, 16(11), 646–655. 10.1016/S0169-5347(01)02283-2

[ece36255-bib-0033] Díaz, S. , Kattge, J. , Cornelissen, J. H. C. , Wright, I. J. , Lavorel, S. , Dray, S. , … Gorné, L. D. (2016). The global spectrum of plant form and function. Nature, 529(7585), 167–171. 10.1038/nature16489 26700811

[ece36255-bib-0034] Dormann, C. F. , Elith, J. , Bacher, S. , Buchmann, C. , Carl, G. , Carré, G. , … Lautenbach, S. (2013). Collinearity: A review of methods to deal with it and a simulation study evaluating their performance. Ecography, 36(1), 027–046. 10.1111/j.1600-0587.2012.07348.x

[ece36255-bib-0035] Doyle, J. , & Doyle, J. L. (1987). Genomic plant DNA preparation from fresh tissue‐CTAB method. Phytochemical Bulletin, 19(11), 11–15.

[ece36255-bib-0036] Dwyer, J. , Hobbs, R. , & Mayfield, M. (2014). Specific leaf area responses to environmental gradients through space and time. Ecology, 95(2), 399–410. 24669733 10.1890/13-0412.1

[ece36255-bib-0037] Earl, D. A. , & vonHoldt, B. M. (2012). STRUCTURE HARVESTER: A website and program for visualizing STRUCTURE output and implementing the Evanno method. Conservation Genetics Resources, 4(2), 359–361. 10.1007/s12686-011-9548-7

[ece36255-bib-0038] Ellegren, H. (2004). Microsatellites: Simple sequences with complex evolution. Nature Reviews Genetics, 5(6), 435–445. 10.1038/nrg1348 15153996

[ece36255-bib-0039] Farooq, M. , Wahid, A. , Fujita, K. D. , & Basra, S. M. A. (2009). Plant drought stress: Effects, mechanisms and management. Agronomy for Sustainable Development, 29, 185–212. 10.1051/agro

[ece36255-bib-0040] Farquhar, G. D. , Buckley, T. N. , & Miller, J. M. (2002). Optimal stomatal control in relation to leaf area and nitrogen content. Silva Fennica, 36(3), 625–637. 10.14214/sf.530

[ece36255-bib-0041] Freeland, J. R. , Kirk, H. , & Petersen, S. D. (2011). Molecular ecology (2nd edition). Hoboken, NJ: John Wiley & Sons 10.1002/9780470979365

[ece36255-bib-0042] Garve, E. (2004). Rote Liste und Florenliste der Farn‐und Blütenpflanzen in Niedersachsen und Bremen. Hannover, Germany: Niedersächsisches Landesamt für Ökologie‐Fachbehörde für Naturschutz.

[ece36255-bib-0043] Gaudet, C. L. , & Keddy, P. A. (1988). A comparative approach to predicting competitive ability from plant traits. Nature, 334(6179), 242–243. 10.1038/334242a0

[ece36255-bib-0044] Geber, M. A. , & Griffen, L. R. (2003). Inheritance and natural selection on functional traits. International Journal of Plant Sciences, 164(S3), S21–S42. 10.1086/368233

[ece36255-bib-0045] Ghalambor, C. K. , McKay, J. K. , Carroll, S. P. , & Reznick, D. N. (2007). Adaptive versus non‐adaptive phenotypic plasticity and the potential for contemporary adaptation in new environments. Functional Ecology, 21(3), 394–407. 10.1111/j.1365-2435.2007.01283.x

[ece36255-bib-0046] Givnish, T. J. (1987). Comparative studies of leaf form: Assessing the relative roles of selective pressures and phylogenetic constraints. New Phytologist, 106, 131–160. 10.1111/j.1469-8137.1987.tb04687.x

[ece36255-bib-0047] Gram, W. K. , & Sork, V. L. (2001). Association between environmental and genetic heterogeneity in forest tree populations. Ecology, 82(7), 2012–2021. 10.2307/2680065

[ece36255-bib-0048] Gratani, L. (2014). Plant phenotypic plasticity in response to environmental factors. Advances in Botany, 2014, 1–17. 10.1155/2014/208747

[ece36255-bib-0049] Griffin, K. L. , Epstein, D. J. , & Boelman, N. T. (2013). Hill slope variations in chlorophyll fluorescence indices and leaf traits in a small arctic watershed. Arctic, Antarctic, and Alpine Research, 45(1), 39–49. 10.1657/1938-4246-45.1.39

[ece36255-bib-0050] Hahn, T. , Kettle, C. J. , Ghazoul, J. , Hennig, E. I. , & Pluess, A. R. (2013). Landscape composition has limited impact on local genetic structure in mountain clover, *Trifolium Montanum* L. Journal of Heredity, 104(6), 842–852. 10.1093/jhered/est058 24064981

[ece36255-bib-0051] Hardy, G. H. (1908). Mendelian proportions in a mixed population In Peters J. A. (Ed.), Classic papers in genetics (pp. 60–62). Englewood Cliffs, NJ: Prentice‐Hall Biological Science Series 10.1126/science.28.706.49

[ece36255-bib-0052] Harrell, F. E. (2019). Hmisc ‐ Harrell Miscellaneous. Retrieved fromhttps://github.com/harrelfe/Hmisc

[ece36255-bib-0053] Helm, J. , Dutoit, T. , Saatkamp, A. , Bucher, S. F. , Leiterer, M. , & Römermann, C. (2019). Recovery of Mediterranean steppe vegetation after cultivation: Legacy effects on plant composition, soil properties and functional traits. Applied Vegetation Science, 22(1), 71–84. 10.1111/avsc.12415

[ece36255-bib-0054] Hijmans, R. J. (2016). geosphere: Spherical trigonometry. Retrieved fromhttps://CRAN.R‐project.org/package=geosphere

[ece36255-bib-0055] Hijmans, R. J. , Cameron, S. E. , Parra, J. L. , Jones, P. G. , & Jarvis, A. (2005). Very high resolution interpolated climate surfaces for global land areas. International Journal of Climatology, 25(15), 1965–1978. 10.1002/joc.1276

[ece36255-bib-0056] Huenneke, L. F. (1991). Ecological implications of variation in plant populations In Falk D. A., & Holsinger K. E. (Eds.), Genetics and conservation of rare plants (pp. 31–44). New York, NY: Oxford University Press.

[ece36255-bib-0057] Hufford, K. M. , & Mazer, S. J. (2003). Plant ecotypes: Genetic differentiation in the age of ecological restoration. Trends in Ecology and Evolution, 18(3), 147–155. 10.1016/S0169-5347(03)00002-8

[ece36255-bib-0058] Hughes, P. W. , Soppe, W. J. J. , & Albani, M. C. (2019). Seed traits are pleiotropically regulated by the flowering time gene PERPETUAL FLOWERING 1 (PEP1) in the perennial *Arabis alpina* . Molecular Ecology, 28(5), 1183–1201. 10.1111/mec.15034 30712274 PMC6850658

[ece36255-bib-0059] Jäger, E. (2011). *Trifolium montanum* L(20th edition). Heidelberg, Germany: Springer Spektrum.

[ece36255-bib-0060] Jakobsson, M. , & Rosenberg, N. A. (2007). CLUMPP: A cluster matching and permutation program for dealing with label switching and multimodality in analysis of population structure. Bioinformatics, 23(14), 1801–1806. 10.1093/bioinformatics/btm233 17485429

[ece36255-bib-0061] Jaleel, C. A. , Manivannan, P. , Wahid, A. , Farooq, M. , Al‐Juburi, H. J. , Somasundaram, R. , & Panneerselvam, R. (2009). Drought stress in plants: A review on morphological characteristics and pigments composition. International Journal of Agriculture & Biology, 11, 100–105.

[ece36255-bib-0062] Jombart, T. , Kamvar, Z. N. , Collins, C. , Lustrik, R. , Beugin, M.‐P. , Knaus, B. J. , & Jombart, M. T. (2018). adegenet: A R package for the multivariate analysis of genetic markers. Retrieved fromhttps://github.com/thibautjombart/adegenet 10.1093/bioinformatics/btn129 18397895

[ece36255-bib-0063] Kalinowski, S. T. (2004). Counting alleles with rarefaction: Private alleles and hierarchical sampling designs. Conservation Genetics, 5(4), 539–543. 10.1023/B:COGE.0000041021.91777.1a

[ece36255-bib-0064] Karbstein, K. (2020). Saturation‐of‐diversity‐variables. Github Repositoryhttps://github.com/KK260/saturation‐of‐diversity‐variables

[ece36255-bib-0065] Karbstein, K. , Tomasello, S. , & Prinz, K. (2019). Desert‐like badlands and surrounding (semi‐)dry grasslands of Central Germany promote small‐scale phenotypic and genetic differentiation in *Thymus praecox* . Ecology and Evolution, 9, 14066–14084. 10.1002/ece3.5844 31938504 PMC6953696

[ece36255-bib-0066] Kattge, J. , Bönisch, G. , Díaz, S. , Lavorel, S. , Prentice, I. C. , Leadley, P. , … Wirth, C. (2020). TRY plant trait database ‐ enhanced coverage and open access. Global Change Biology, 26(1), 119–188. 10.1111/gcb.14904 31891233

[ece36255-bib-0067] Kattge, J. , Díaz, S. , Lavorel, S. , Prentice, I. C. , Leadley, P. , Bönisch, G. , … Wirth, C. (2011). TRY ‐ a global database of plant traits. Global Change Biology, 17(9), 2905–2935. 10.1111/j.1365-2486.2011.02451.x

[ece36255-bib-0068] Kerkhoff, A. J. , Fagan, W. F. , Elser, J. J. , & Enquist, B. J. (2006). Phylogenetic and growth form variation in the scaling of nitrogen and phosphorus in the seed plants. The American Naturalist, 168(4), E103–E122. 10.1086/507879 17004214

[ece36255-bib-0069] König, P. , Tautenhahn, S. , Cornelissen, J. C. , Kattge, J. , Bönisch, G. , & Römermann, C. (2018). Advances in flowering phenology across the Northern Hemisphere are explained by functional traits. Global Ecology and Biogeography, 27(3), 310–321. 10.1111/geb.12696

[ece36255-bib-0070] Korbie, D. J. , & Mattick, J. S. (2008). Touchdown PCR for increased specificity and sensitivity in PCR amplification. Nature Protocols, 3(9), 1452–1456. 10.1038/nprot.2008.133 18772872

[ece36255-bib-0071] Lande, R. (2009). Adaptation to an extraordinary environment by evolution of phenotypic plasticity and genetic assimilation. Journal of Evolutionary Biology, 22(7), 1435–1446. 10.1111/j.1420-9101.2009.01754.x 19467134

[ece36255-bib-0072] Leimu, R. , Mutikainen, P. , Koricheva, J. , & Fischer, M. (2006). How general are positive relationships between plant population size, fitness and genetic variation? Journal of Ecology, 94(5), 942–952. 10.1111/j.1365-2745.2006.01150.x

[ece36255-bib-0073] Li, Y. C. , Korol, A. B. , Fahima, T. , Beiles, A. , & Nevo, E. (2002). Microsatellites: Genomic distribution, putative functions and mutational mechanisms: A review. Molecular Ecology, 11(12), 2453–2465. 10.1046/j.1365-294X.2002.01643.x 12453231

[ece36255-bib-0074] Linhardt, Y. B. , & Grant, M. C. (1996). Evolutionary significance of local genetic differentiation in plants. Annual Review of Ecology and Systematics, 27, 237–277. 10.1146/annurev.soc.29.010202.100030

[ece36255-bib-0075] Locascio, A. , Lucchin, M. , & Varotto, S. (2009). Characterization of a MADS FLOWERING LOCUS C‐LIKE (MFL) sequence in *Cichorium intybus*: A comparative study of CiMFL and AtFLC reveals homologies and divergences in gene function. New Phytologist, 182(3), 630–643. 10.1111/j.1469-8137.2009.02791.x 19291007

[ece36255-bib-0076] MacArthur, R. , & Wilson, E. (1967). The theory of island biogeography. Princeton, NJ: Princeton University Press.

[ece36255-bib-0077] Matter, P. , Kettle, C. J. , Ghazoul, J. , Hahn, T. , & Pluess, A. R. (2013). Evaluating contemporary pollen dispersal in two common grassland species *Ranunculus bulbosus* L. (Ranunculaceae) and *Trifolium montanum* L. (Fabaceae) using an experimental approach. Plant Biology, 15(3), 583–592. 10.1111/j.1438-8677.2012.00667.x 23016803

[ece36255-bib-0078] Matter, P. , Määttänen, K. , Kettle, C. J. , Ghazoul, J. , & Pluess, A. R. (2012). Eleven microsatellite markers for the mountain clover *Trifolium montanum* (Fabaceae). American Journal of Botany, 99(11), 447–449. 10.3732/ajb.1200102 23108463

[ece36255-bib-0079] Maxwell, K. , & Johnson, G. N. (2000). Chlorophyll fluorescence – A practical guide. Journal of Experimental Botany, 51(345), 659–668. 10.1093/jxb/51.345.659 10938857

[ece36255-bib-0080] Meusel, H. , & Jäger, E. (1998). Vergleichende Chorologie der Zentraleuropäischen Flora‐Band I, II, III. Jena, DE: Gustav Fischer Verlag.

[ece36255-bib-0081] Moles, A. T. , Warton, D. I. , Warman, L. , Swenson, N. G. , Laffan, S. W. , Zanne, A. E. , … Leishman, M. R. (2009). Global patterns in plant height. Journal of Ecology, 97(5), 923–932. 10.1111/j.1365-2745.2009.01526.x

[ece36255-bib-0082] Moran, P. A. P. (1950). Notes on continuous stochastic phenomena. Biometrika Trust, 37(1–2), 17–23. 10.2307/2332142 15420245

[ece36255-bib-0083] Nei, M. (1973). Analysis of gene diversity in subdivided populations. Proc Nat Acad Sci USA, 70(12), 3321–3323. 10.1073/pnas.70.12.3321 4519626 PMC427228

[ece36255-bib-0084] Nei, M. (1978). Estimation of average heterozygosity and genetic distance from a small number of individuals. Genetics, 89(3), 583–590. 10.3390/ijms15010277 17248844 PMC1213855

[ece36255-bib-0085] Nicotra, A. B. , Atkin, O. K. , Bonser, S. P. , Davidson, A. M. , Finnegan, E. J. , Mathesius, U. , … van Kleunen, M. (2010). Plant phenotypic plasticity in a changing climate. Trends in Plant Science, 15(12), 684–692. 10.1016/j.tplants.2010.09.008 20970368

[ece36255-bib-0086] Nicotra, A. B. , Segal, D. L. , Hoyle, G. L. , Schrey, A. W. , Verhoeven, K. J. F. , & Richards, C. L. (2015). Adaptive plasticity and epigenetic variation in response to warming in an Alpine plant. Ecology and Evolution, 5(3), 634–647. 10.1002/ece3.1329 25691987 PMC4328768

[ece36255-bib-0087] Nock, C. A. , Vogt, R. J. , & Beisner, B. E. (2016). Functional traits. ELS, February, 1–8. 10.1002/9780470015902.a0026282

[ece36255-bib-0088] Paillotin, G. (1976). Movement of excitations in the photosynthetic domains of photosystem II. Journal of Theoretical Biology, 58(1), 237–252. 10.1016/0022-5193(76)90150-8 957686

[ece36255-bib-0089] Peakall, R. , & Smouse, P. E. (2006). GenAlEx 6: Genetic analysis in Excel. Population genetic software for teaching and research. Molecular Ecology Notes, 6(1), 288–295. 10.1111/j.1471-8286.2005.01155.x PMC3463245 22820204

[ece36255-bib-0090] Peakall, R. , & Smouse, P. E. (2012). GenAlEx 6.5: Genetic analysis in Excel. Population genetic software for teaching and research – An update. Bioinformatics, 28(19), 2537–2539. 10.1093/bioinformatics/bts460 22820204 PMC3463245

[ece36255-bib-0091] Pérez‐Harguindeguy, N. , Díaz, S. , Garnier, E. , Lavorel, S. , Poorter, H. , Jaureguiberry, P. , … Cornelissen, J. H. C. (2013). New handbook for standardised measurement of plant functional traits worldwide. Australian Journal of Botany, 61(3), 167–234. 10.1071/BT12225

[ece36255-bib-0092] Petchey, O. L. , & Gaston, K. J. (2006). Functional diversity: Back to basics and looking forward. Ecology Letters, 9(6), 741–758. 10.1111/j.1461-0248.2006.00924.x 16706917

[ece36255-bib-0093] Prinz, K. , Weising, K. , & Hensen, I. (2009). Genetic structure of coastal and inland populations of the annual halophyte *Suaeda maritima* (L.) dumort. in Central Europe, inferred from amplified fragment length polymorphism markers. Plant Biology, 11(6), 812–820. 10.1111/j.1438-8677.2008.00178.x 19796358

[ece36255-bib-0094] Pritchard, J. K. , Stephens, M. , & Donnelly, P. (2000). Inference of population structure using multilocus genotype data. Genetics, 155(2), 945–959. 10.1111/j.1471-8286.2007.01758.x 10835412 PMC1461096

[ece36255-bib-0095] Ramalho, J. C. , Rebelo, M. C. , Santos, M. E. , Antunes, M. L. , & Nunes, M. A. (1995). Effects of calcium deficiency on *Coffea arabica*. Nutrient changes and correlation of calcium levels with some photosynthetic parameters. Plant and Soil, 172(1), 87–96. 10.1007/BF00020862

[ece36255-bib-0096] Razaq, M. , Zhang, P. , Shen, H.‐L. , & Salahuddin, (2017). Influence of nitrogen and phosphorous on the growth and root morphology of *Acer mono* . PLoS ONE, 12(2), e0171321 10.1371/journal.pone.0171321 28234921 PMC5325205

[ece36255-bib-0097] Reed, D. H. , & Frankham, R. (2003). Correlation between fitness and genetic diversity. Conservation Biology, 17(1), 230–237. 10.1046/j.1523-1739.2003.01236.x

[ece36255-bib-0098] Reich, P. B. , & Hobbie, S. E. (2013). Decade‐long soil nitrogen constraint on the CO_2_ fertilization of plant biomass. Nature Climate Change, 3(3), 278–282. 10.1038/nclimate1694

[ece36255-bib-0099] Reisch, C. , & Bernhardt‐Römermann, M. (2014). The impact of study design and life history traits on genetic variation of plants determined with AFLPs. Plant Ecology, 215(12), 1493–1511. 10.1007/s11258-014-0409-9

[ece36255-bib-0100] Reisch, C. , & Schmid, C. (2019). Species and genetic diversity are not congruent in fragmented dry grasslands. Ecology and Evolution, 9(1), 664–671. 10.1002/ece3.4791 30680146 PMC6342089

[ece36255-bib-0101] Reusch, T. B. , Ehlers, A. , Hämmerli, A. , & Worm, B. (2005). Ecosystem recovery after climatic extremes enhanced by genotypic diversity. Proceedings of the National Academy of Sciences of the United States of America, 102(8), 2826–2831. 10.1073/pnas.0500008102 15710890 PMC549506

[ece36255-bib-0102] Rice, A. , Glick, L. , Abadi, S. , Einhorn, M. , Kopelman, N. M. , Salman‐Minkov, A. , … Mayrose, I. (2015). The Chromosome Counts Database (CCDB) – A community resource of plant chromosome numbers. New Phytologist, 206(1), 19–26. 10.1111/nph.13191 25423910

[ece36255-bib-0103] Römermann, C. , Bernhardt‐Römermann, M. , Kleyer, M. , & Poschlod, P. (2009). Substitutes for grazing in semi‐natural grasslands – Do mowing or mulching represent valuable alternatives to maintain vegetation structure? Journal of Vegetation Science, 20(6), 1086–1098.

[ece36255-bib-0104] Römermann, C. , Bucher, S. F. , Hahn, M. , & Bernhardt‐Römermann, M. (2016). Plant functional traits ‐ fixed facts or variable depending on the season? Folia Geobotanica, 51(2), 143–159. 10.1007/s12224-016-9250-3

[ece36255-bib-0105] Rosenberg, N. A. (2004). DISTRUCT: A program for the graphical display of population structure. Molecular Ecology Notes, 4(1), 137–138. 10.1046/j.1471-8286.2003.00566.x

[ece36255-bib-0106] Sack, L. , Cowan, P. D. , Jaikumar, N. , & Holbrook, N. M. (2003). The ‘hydrology’ of leaves: Co‐ordination of structure and function in temperate woody species. Plant, Cell and Environment, 26(8), 1343–1356. 10.1046/j.0016-8025.2003.01058.x

[ece36255-bib-0107] Saghai‐Maroof, M. A. , Soliman, K. M. , Jorgensen, R. A. , & Allard, R. W. (1984). Ribosomal DNA spacer‐length polymorphisms in barley: Mendelian inheritance, chromosomal location, and population dynamics. Proceedings of the National Academy of Sciences of the United States of America, 81(24), 8014–8018. 10.1073/pnas.81.24.8014 6096873 PMC392284

[ece36255-bib-0108] Sakaguchi, S. , Horie, K. , Ishikawa, N. , Nishio, S. , Worth, J. R. , Fukushima, K. , … Ito, M. (2019). Maintenance of soil ecotypes of *Solidago virgaurea* in close parapatry via divergent flowering time and selection against immigrants. Journal of Ecology, 107(1), 418–435. 10.1111/1365-2745.13034

[ece36255-bib-0109] Schleuning, M. , & Matthies, D. (2008). Habitat change and plant demography: Assessing the extinction risk of a formerly common grassland perennial. Conservation Biology, 23(1), 174–183. 10.1111/j.1523-1739.2008.01054.x 18847437

[ece36255-bib-0110] Schleuning, M. , Niggemann, M. , Becker, U. , & Matthies, D. (2009). Negative effects of habitat degradation and fragmentation on the declining grassland plant *Trifolium montanum* . Basic and Applied Ecology, 10(1), 61–69. 10.1016/j.baae.2007.12.002

[ece36255-bib-0111] Schweiger, A. K. , Cavender‐Bares, J. , Townsend, P. A. , Hobbie, S. E. , Madritch, M. D. , Wang, R. , … Gamon, J. A. (2018). Plant spectral diversity integrates functional and phylogenetic components of biodiversity and predicts ecosystem function. Nature Ecology and Evolution, 2(6), 976–982. 10.1038/s41559-018-0551-1 29760440

[ece36255-bib-0112] GBIF Secretariat . (2017). *Trifolium montanum* L. GBIF Backbone Taxonomy. Checklist Dataset. Retrieved from 10.15468/39omei

[ece36255-bib-0113] Shannon, C. E. (1948). A mathematical theory of communication. The Bell System Technical Journal, 27, 379–423. 10.1145/584091.584093

[ece36255-bib-0114] Simpson, E. H. (1949). Measurement of diversity. Nature, 163(4148), 688 10.1038/163688a0

[ece36255-bib-0115] Sims, L. , Pastor, J. , Lee, T. , & Dewey, B. (2012). Nitrogen, phosphorus and light effects on growth and allocation of biomass and nutrients in wild rice. Oecologia, 170(1), 65–76. 10.1007/s00442-012-2296-x 22407062

[ece36255-bib-0116] Strasser, R. J. , Srivastava, A. , & Tsimilli‐Michael, M. (2000). The fluorescence transient as a tool to characterize and screen photosynthetic samples In Yunus M., Pathre U., & Mohanty P. (Eds.), Probing photosynthesis: Mechanisms, regulation and adaptation (pp. 445–483). London, UK and New York, NY: Taylor and Francis.

[ece36255-bib-0117] Strasser, R. J. , Tsimilli‐Michael, M. , & Srivastava, A. (2004). Analysis of the chlorophyll a fluorescence transient In Papageorgiou G. C. & Govindjee (Eds.), Chlorophyll a fluorescence (2nd edition, pp. 321–362). Dordrecht, NL: Springer 10.1007/978-1-4020-3218-9_12

[ece36255-bib-0118] Sultan, S. E. (2000). Phenotypic plasticity for plant development, function and life history. Trends in Plant Science, 5, 537–542. 10.1016/S1360-1385(00)01797-0 11120476

[ece36255-bib-0119] Szczecińska, M. , Sramko, G. , Wołosz, K. , & Sawicki, J. (2016). Genetic diversity and population structure of the rare and endangered plant species *Pulsatilla patens* (L.) Mill in East Central Europe. PLoS ONE, 11(3), 1–24. 10.1371/journal.pone.0151730 PMC4803199 27003296

[ece36255-bib-0120] Taiyun, W. , & Simko, V. (2017). corrplot ‐ visualization of a correlation matrix. Retrieved fromhttps://github.com/taiyun/corrplot

[ece36255-bib-0121] Tautenhahn, S. , Grün‐Wenzel, C. , Jung, M. , Higgins, S. , & Römermann, C. (2019). On the relevance of intraspecific trait variability ‐ A synthesis of 56 dry grassland sites across Europe. Flora, 254, 161–172. 10.1016/j.flora.2019.03.002

[ece36255-bib-0122] Thornsberry, J. M. , Goodman, M. M. , Doebley, J. , Kresovich, S. , Nielsen, D. , & Buckler, E. S. (2001). Dwarf8 polymorphisms associate with variation in flowering time. Nature Genetics, 28(3), 286–289. 10.1038/90135 11431702

[ece36255-bib-0123] Tilman, D. (2001). Functional diversity. Encyclopedia of Biodiversity, 2, 587‐596. 10.1016/B978-0-12-384719-5.00061-7

[ece36255-bib-0124] Vellend, M. , & Geber, M. A. (2005). Connections between species diversity and genetic diversity. Ecology Letters, 8(7), 767–781. 10.1111/j.1461-0248.2005.00775.x

[ece36255-bib-0125] Via, S. , Gomulkiewicz, R. , De Jong, G. , Scheiner, S. M. , Schlichting, C. D. , & Van Tienderen, P. H. (1995). Adaptive phenotypic plasticity: Consensus and controversy. Trends in Ecology & Evolution, 10(5), 212–217. 10.1016/S0169-5347(00)89061-8 21237012

[ece36255-bib-0126] Vieira, M. L. C. , Santini, L. , Diniz, A. L. , & Munhoz, C. D. F. (2016). Microsatellite markers: What they mean and why they are so useful. Genetics and Molecular Biology, 39(3), 312–328. 10.1590/1678-4685-GMB-2016-0027 27561112 PMC5004837

[ece36255-bib-0127] Violle, C. , Enquist, B. J. , McGill, B. J. , Jiang, L. , Albert, C. H. , Hulshof, C. , … Messier, J. (2012). The return of the variance: Intraspecific variability in community ecology. Trends in Ecology & Evolution, 27(4), 244–252. 10.1016/j.tree.2011.11.014 22244797

[ece36255-bib-0128] Violle, C. , Navas, M. , Vile, D. , Kazakou, E. , Fortunel, C. , Hummel, I. , & Garnier, E. (2007). Let the concept of trait be functional!. Oikos, 116(5), 882–892. 10.1111/j.2007.0030-1299.15559.x

[ece36255-bib-0129] Vittoz, P. , & Engler, R. (2007). Seed dispersal distances: A typology based on dispersal modes and plant traits. Botanica Helvetica, 117, 109–124.

[ece36255-bib-0130] Waitt, D. E. , & Levin, D. A. (1998). Genetic and phenotypic correlations in plants: A botanical test of Cheverud’s conjecture. Heredity, 80(3), 310–319. 10.1046/j.1365-2540.1998.00298.x

[ece36255-bib-0131] Weinberg, W. (1908). Über Vererbungsgesetze beim Menschen. Zeitschrift Für Induktive Abstammungs‐ Und Vererbungslehre, 1(1), 377–392. 10.1007/BF01990605

[ece36255-bib-0132] Weinhold, B. (2006). Epigenetics: The science of change. Environmental Health Perspectives, 114(3), 160–167. 10.1289/ehp.114-a160 PMC1392256 16507447

[ece36255-bib-0133] Wickham, H. , & Ruiz, E. (2019). A ‘dplyr’ back end for databases.

[ece36255-bib-0134] Wood, C. M. , McKinney, S. T. , & Loftin, C. S. (2017). Intraspecific functional diversity of common species enhances community stability. Ecology and Evolution, 7(5), 1553–1560. 10.1002/ece3.2721 28261464 PMC5330891

[ece36255-bib-0135] Woodward, F. I. , Lake, J. A. , & Quick, W. P. (2002). Stomatal development and CO_2_: Ecological consequences. New Phytologist, 153(3), 477–484. 10.1046/j.0028-646X.2001.00338.x 33863227

[ece36255-bib-0136] Wright, K. (2018). corrgram: Plot a correlogram. Retrieved from https://cran.r‐project.org

