# Supplementary material for: Plant intraspecific functional trait variation is related to within‐habitat heterogeneity and genetic diversity in Trifolium montanum L
Source: Ecol Evol. 2020 Apr 16;10(11):5015–33. doi: 10.1002/ece3.6255 (PMC7297743; doi:10.1002/ece3.6255)
Supplement: Supplementary file 1 — Supplementary Material [file ECE3-10-5015-s001.docx]

**Appendix**

**Plant intraspecific functional trait variation is related to within-habitat heterogeneity and genetic diversity in *Trifolium montanum* L.**

**Karbstein, Kevin^1,4,*^, Prinz, Kathleen^1,2^, Hellwig, Frank^1^ & Römermann, Christine^1,3^**

1. Friedrich-Schiller-University Jena, Institute of Ecology and Systematics, Philosophenweg 16, D-07743 Jena, Germany
2. Landschaftspflegeverband Suedharz/Kyffhaeuser e.V., Uthleber Straße 24, D-99734 Nordhausen, Germany
3. German Centre for Integrative Biodiversity Research (iDiv) Halle-Jena-Leipzig, Deutscher Platz 5e, D-04103 Leipzig, Germany

**4 current working address:** University of Goettingen, Albrecht-von-Haller Institute for Plant Sciences, Department of Systematics, Biodiversity and Evolution of Plants (with Herbarium), Untere Karspuele 2, D-37073, Goettingen, Germany

*** corresponding author**: kevin.karbstein@uni-goettingen.de

|  |  | **estimate** | **standard error** | **t value** | **p value** | **F** | **df** | **R_adj_^2^** |
| --- | --- | --- | --- | --- | --- | --- | --- | --- |
|  |  |  |  |  |  |  |  |  |
| **iFD_CV_** |  |  |  |  |  |  |  |  |
|  | intercept | 0.2027 | 0.0044 | 46.429 | *** |  |  |  |
|  | HD | 0.0234 | 0.0050 | 4.662 | *** | 21.66 | 2 | 0.77 |
|  | H_e_ (GD) | 0.0113 | 0.0050 | 2.253 | * |  |  |  |
|  |  |  |  |  |  |  |  |  |
|  |  |  |  |  |  |  |  |  |
| **RH** |  |  |  |  |  |  |  |  |
|  | intercept | -2.3766 | 0.1538 | -15.452 | *** |  |  |  |
|  | HD | 3.2564 | 0.6036 | 5.395 | *** | 29.1 | 11 | 0.70 |
| **AGB** |  |  |  |  |  |  |  |  |
|  | intercept | -1.2198 | 0.1658 | -7.357 | *** |  |  |  |
|  | HD | 1.6472 | 0.6507 | 2.531 | * | 6.408 | 11 | 0.31 |
| **LA** |  |  |  |  |  |  |  |  |
|  | intercept | -1.4627 | 0.2346 | -6.235 | *** |  |  |  |
|  | HD | 1.4752 | 0.9207 | 1.602 | 0.137 | 2.567 | 11 | 0.12 |
| **SLA** |  |  |  |  |  |  |  |  |
|  | intercept | -2.6304 | 0.1562 | -16.841 | *** |  |  |  |
|  | HD | 1.6106 | 0.6130 | 2.627 | * | 6.903 | 11 | 0.33 |
| **LDMC** |  |  |  |  |  |  |  |  |
|  | intercept | -3.1259 | 0.2030 | -15.395 | *** |  |  |  |
|  | HD | 1.6427 | 0.7969 | 2.061 | ‘ | 4.249 | 11 | 0.21 |
| **F_v_/F_m_** |  |  |  |  |  |  |  |  |
|  | intercept | -4.9311 | 0.2412 | -20.441 | *** |  |  |  |
|  | HD | 2.5067 | 0.9467 | 2.648 | * | 7.01 | 11 | 0.33 |
| **PI** |  |  |  |  |  |  |  |  |
|  | intercept | -1.7246 | 0.2244 | -7.686 | *** |  |  |  |
|  | HD | 2.3337 | 0.8806 | 2.650 | * | 7.023 | 11 | 0.33 |
| **SPS** |  |  |  |  |  |  |  |  |
|  | intercept | -2.1329 | 0.1941 | -10.989 | *** |  |  |  |
|  | HD | -0.1720 | 0.7617 | -0.226 | 0.826 | 0.051 | 11 | 0.00 |
| **SPI** |  |  |  |  |  |  |  |  |
|  | intercept | -1.9396 | 0.1792 | -10.822 | *** |  |  |  |
|  | HD | 0.9324 | 0.7034 | 1.325 | 0.212 | 1.757 | 11 | 0.06 |
|  |  |  |  |  |  |  |  |  |
|  |  |  |  |  |  |  |  |  |
| **RH** |  |  |  |  |  |  |  |  |
|  | intercept | -4.473 | 1.366 | -3.276 | ** |  |  |  |
|  | GD | 4.532 | 2.138 | 2.120 | ‘ | 4.492 | 11 | 0.23 |
| **AGB** |  |  |  |  |  |  |  |  |
|  | intercept | -2.9594 | 0.9524 | -3.107 | ** |  |  |  |
|  | GD | 3.3569 | 1.4912 | 2.251 | * | 5.068 | 11 | 0.25 |
| **LA** |  |  |  |  |  |  |  |  |
|  | intercept | -3.884 | 1.167 | -3.328 | ** |  |  |  |
|  | GD | 4.360 | 1.828 | 2.386 | * | 5.692 | 11 | 0.28 |
| **SLA** |  |  |  |  |  |  |  |  |
|  | intercept | -3.8952 | 0.9792 | -3.978 | ** |  |  |  |
|  | GD | 2.5986 | 1.5332 | 1.695 | 0.118 | 2.873 | 11 | 0.14 |
| **LDMC** |  |  |  |  |  |  |  |  |
|  | intercept | -3.914 | 1.270 | -3.082 | * |  |  |  |
|  | GD | 1.863 | 1.988 | 0.937 | 0.3689 | 0.878 | 11 | 0.00 |
| **F_v_/F_m_** |  |  |  |  |  |  |  |  |
|  | intercept | -5.797 | 1.644 | -3.526 | ** |  |  |  |
|  | GD | 2.316 | 2.574 | 0.900 | 0.387 | 0.810 | 11 | 0.00 |
| **PI** |  |  |  |  |  |  |  |  |
|  | intercept | -2.742 | 1.511 | -1.815 | ‘ |  |  |  |
|  | GD | 2.486 | 2.366 | 1.051 | 0.316 | 1.104 | 11 | 0.00 |
| **SPS** |  |  |  |  |  |  |  |  |
|  | intercept | -2.2656 | 1.0732 | -2.111 | ‘ |  |  |  |
|  | GD | 0.1424 | 1.6804 | 0.085 | 0.934 | 0.007 | 11 | 0.00 |
| **SPI** |  |  |  |  |  |  |  |  |
|  | intercept | -3.1248 | 0.9761 | -3.201 | ** |  |  |  |
|  | GD | 2.2147 | 1.5283 | 1.449 | 0.175 | 2.1 | 11 | 0.08 |

**Table S1** Estimates, standard errors, t values, p values, F, df (degrees of freedom) and R_adj_^2^ values are given for regressions between intraspecific functional trait variation (iFD_CV_) and habitat heterogeneitxy (HD) and genetic diversity indices (N_A_, P_Ap_, H_o_, H_e_ and I), and between particular traits (CV_traits_), and within-habitat heterogeneity (HD) or genetic diversity (H_e_, GD) including 13 T. montanum populations (n = 255 to 260 individuals). Significance levels: *** = p < 0.001, ** = p < 0.01, * = p < 0.05 and ‘ = 0.1 > p > 0.05.


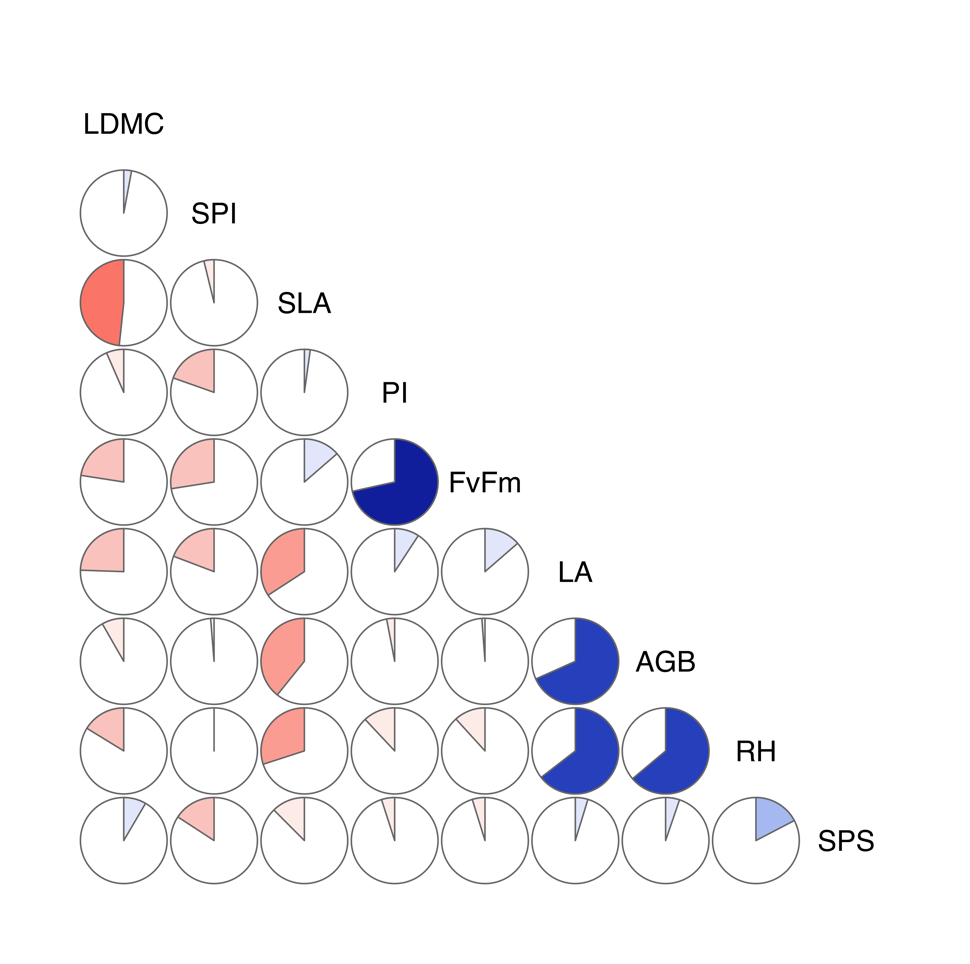


**Fig. S1** Correlations among functional traits based on 260 individuals of 13 T. montanum populations. The value of the Spearman rank coefficient (due to non-normally distributed traits) is represented by the pie chart (blue – negative correlation, red – negative correlation). LDMC = leaf dry matter content, SPI = stomatal pore area index, SLA = specific leaf area, PI = performance index, F_v_/F_m_ = (variable / maximal) fluorescence, LA = leaf area, AGB = total dry aboveground biomass, RH = releasing height, and SPS = stomatal pore surface.

**
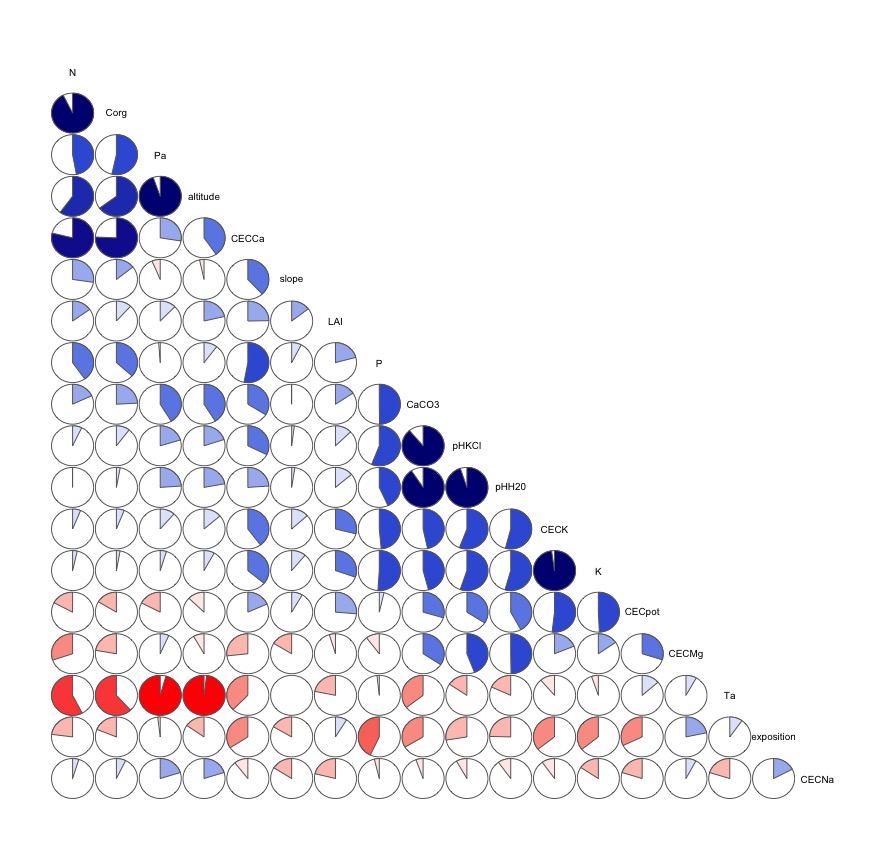
Fig. S2** Correlations among abiotic environmental factors based on 64 replicates of 13 *T. montanum* habitats. The value of the Spearman rank coefficient (due to non-normally distributed traits) is represented by the pie chart (blue – negative correlation, red – negative correlation). N = soil nitrogen content, C_org_ = oragnic carbon content, P_a_ = annual mean precipitation, CEC_Ca, K, Mg, Na_ = soil cation-exchange capacity for calcium, potassium, magnesium, and sodium, LAI = leaf area index, P = soil phosphor content, CaCO_3_ = lime, pH_KCl, H2O_ = soil reaction obtained by potassium chloride or de-ionized water, K = soil potassium content, CEC_pot_ = soil potential cation-exchange capacity.


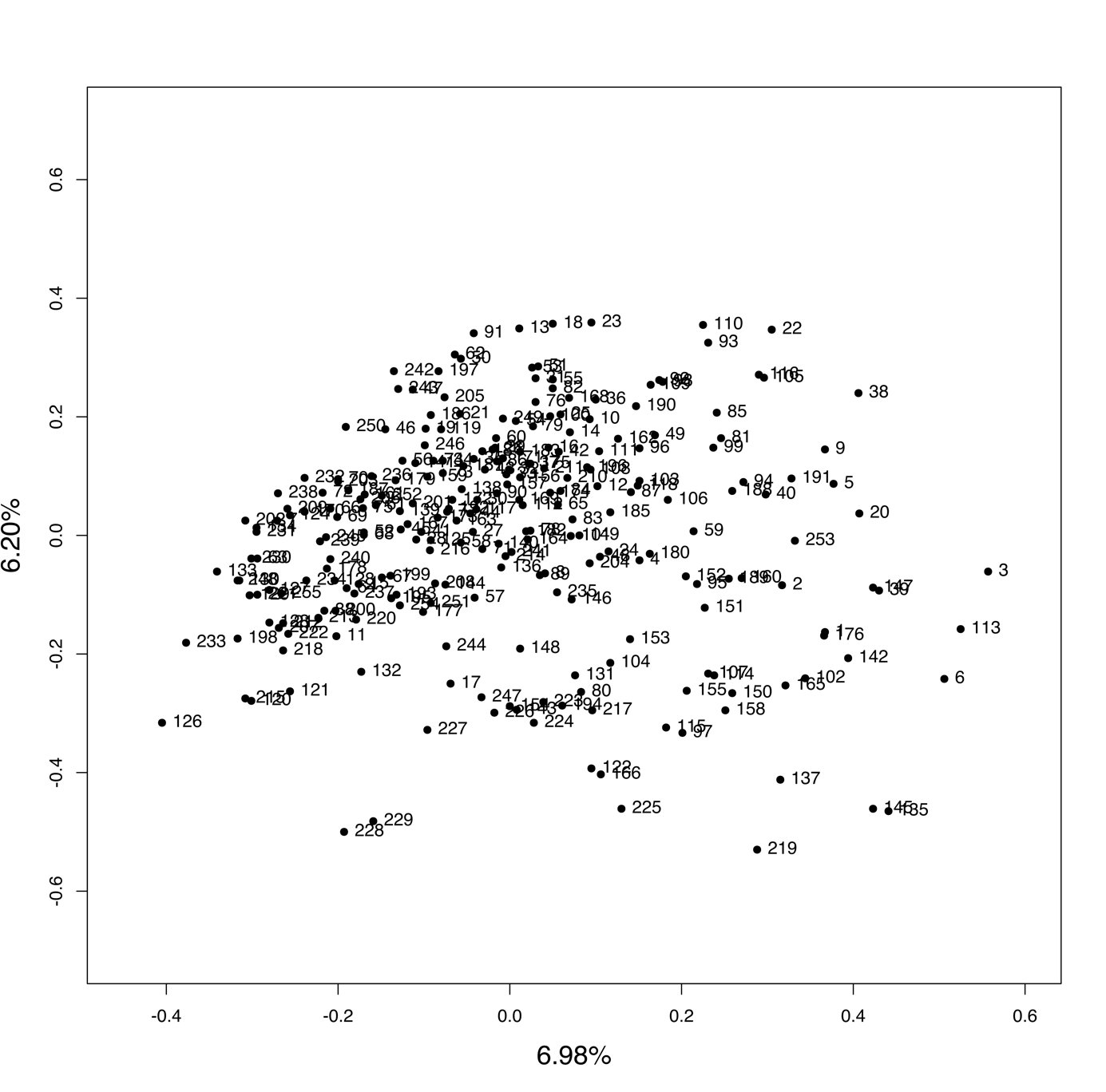
**Fig. S3** Principal coordinate analysis (PCoA) based on Nei´s genetic distances between 255 *T. montanum* individuals belonging to 13 populations. Both axes explained about 13% of the total variation. IDs characterize individuals (see genetic matrix in data repository for details).

**
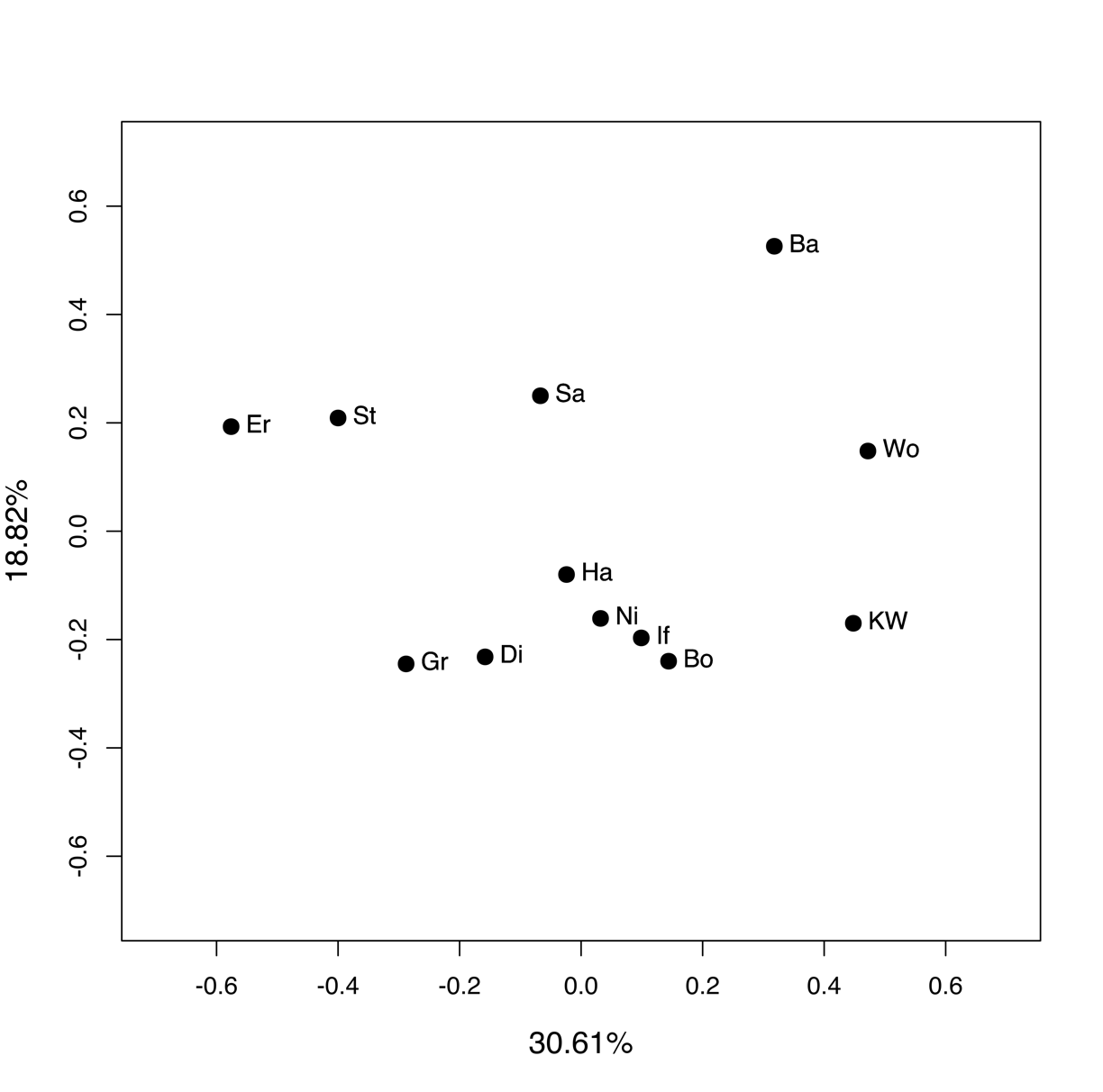
**

**Fig. S4** Principal coordinate analysis (PCoA) based on Nei´s genetic distances between 13 *T. montanum* populations. Both axes explained about 49% of the total variation. See Table 2 for abbreviations.

**
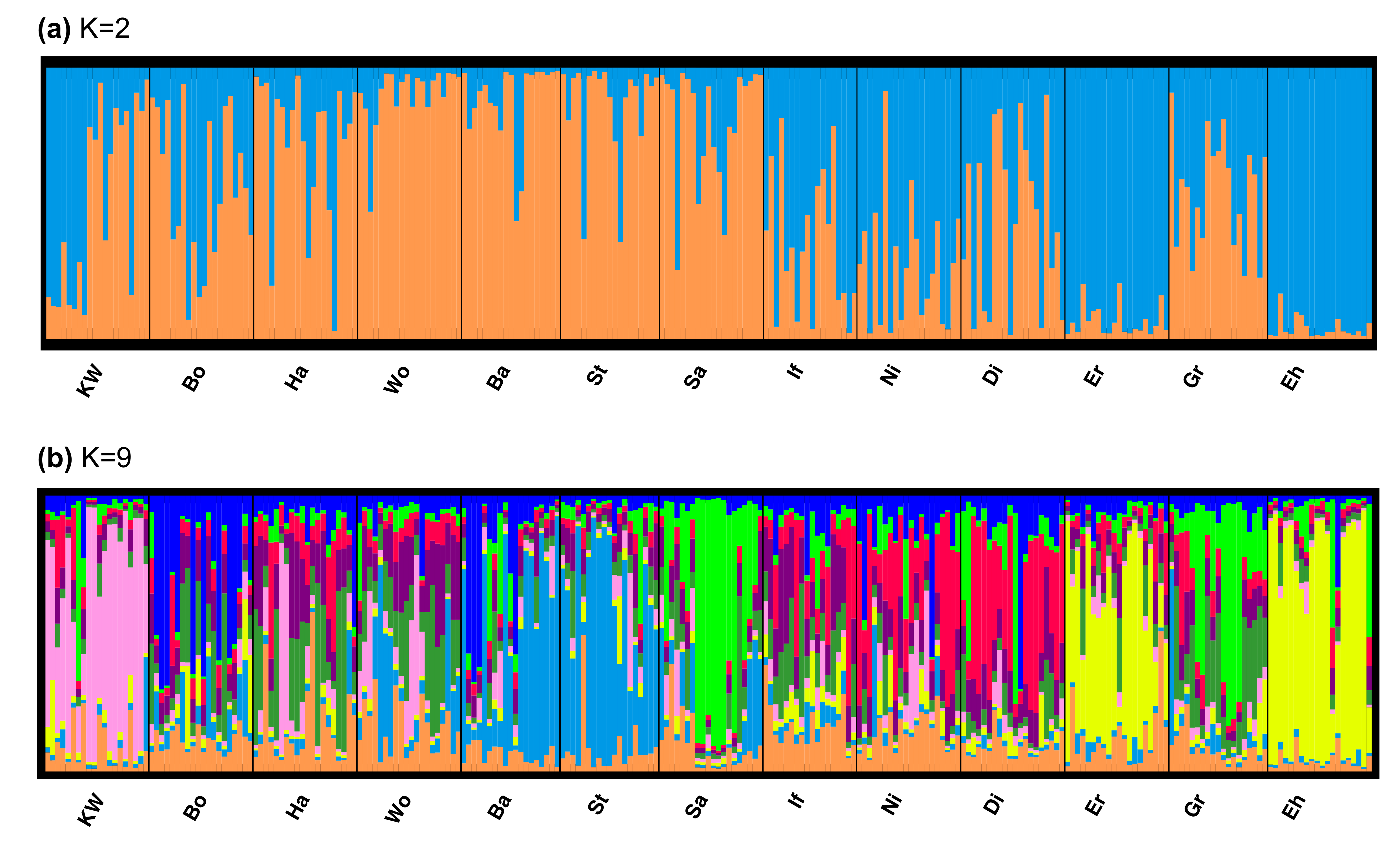
**

**Fig. S5** Barplots illustrating results of STRUCTURE analyses. The Evanno test indicates (a) two (K=2, ∆K=10.01) or (b) nine (K=9, ∆K=17.24) genetic clusters. See Table 2 for abbreviations.


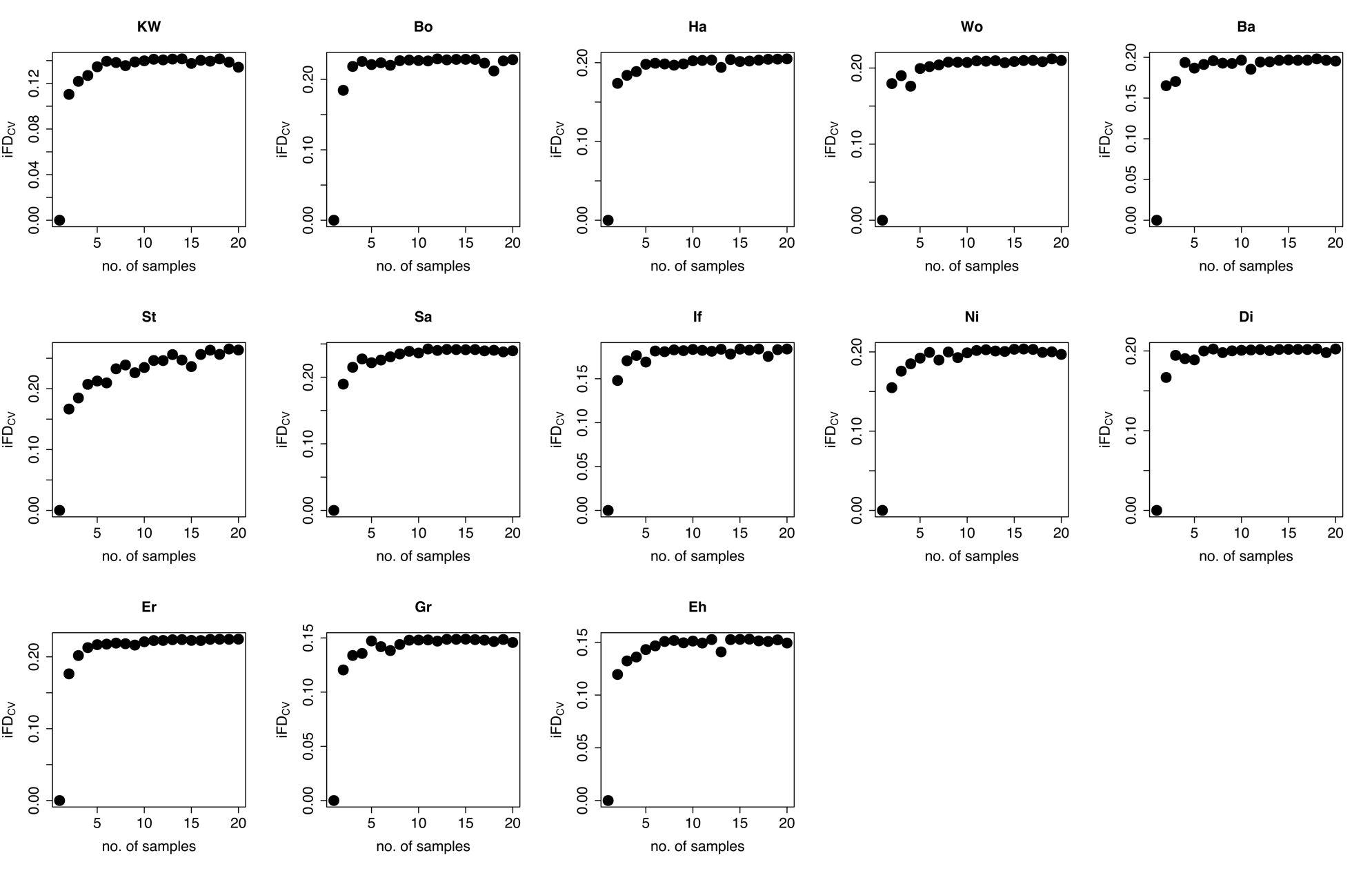


**Fig. S6** Intraspecific functional trait variation (iFD_CV_) per population for different numbers of randomly chosen samples (1-20, with 100 replicates per sample number; see Methods for details).


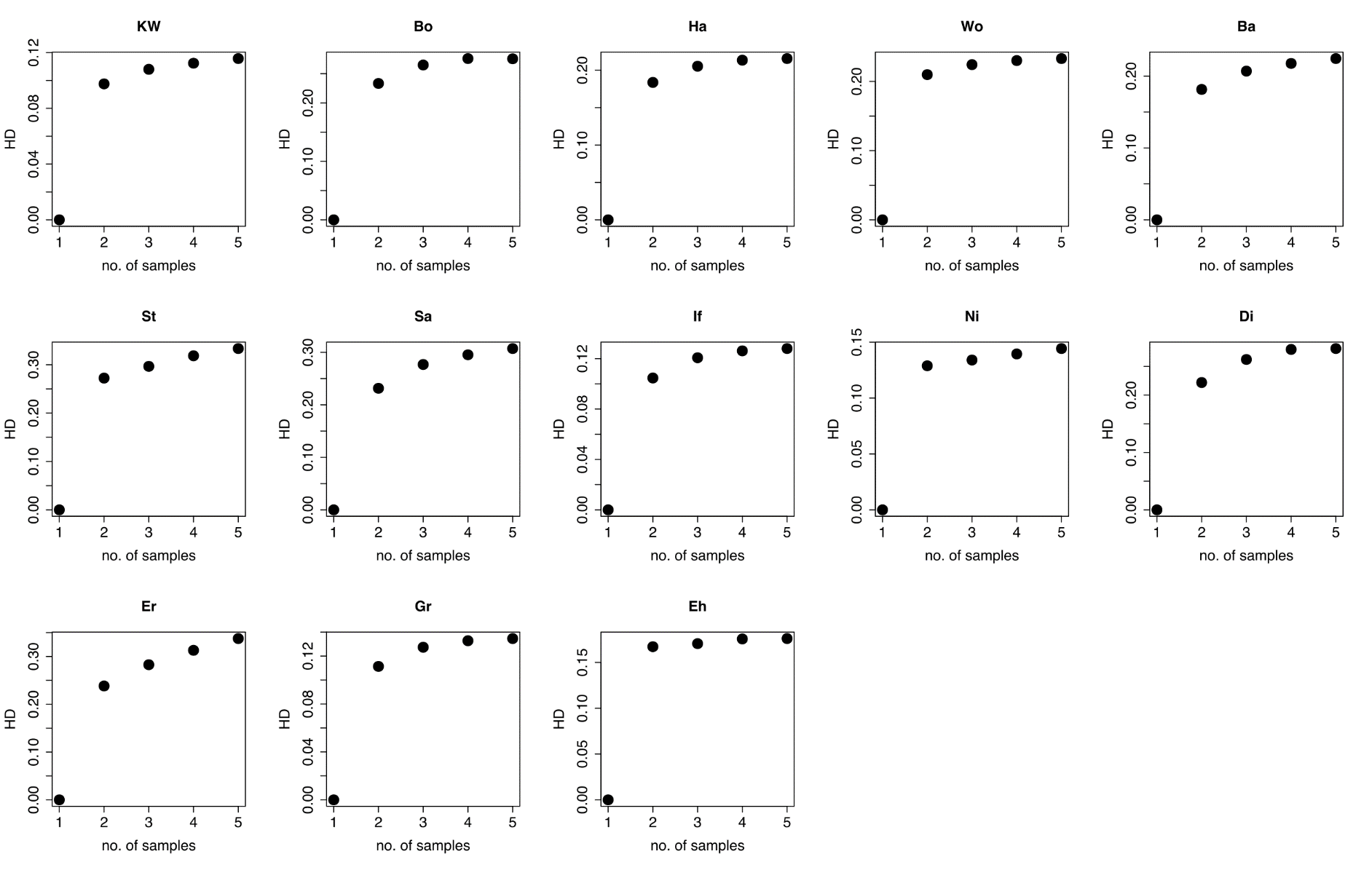


**Fig. S7** Within-habitat heterogeneity (HD) per location for different numbers of randomly chosen samples (1-5, with 100 replicates per sample number; see Methods for details).


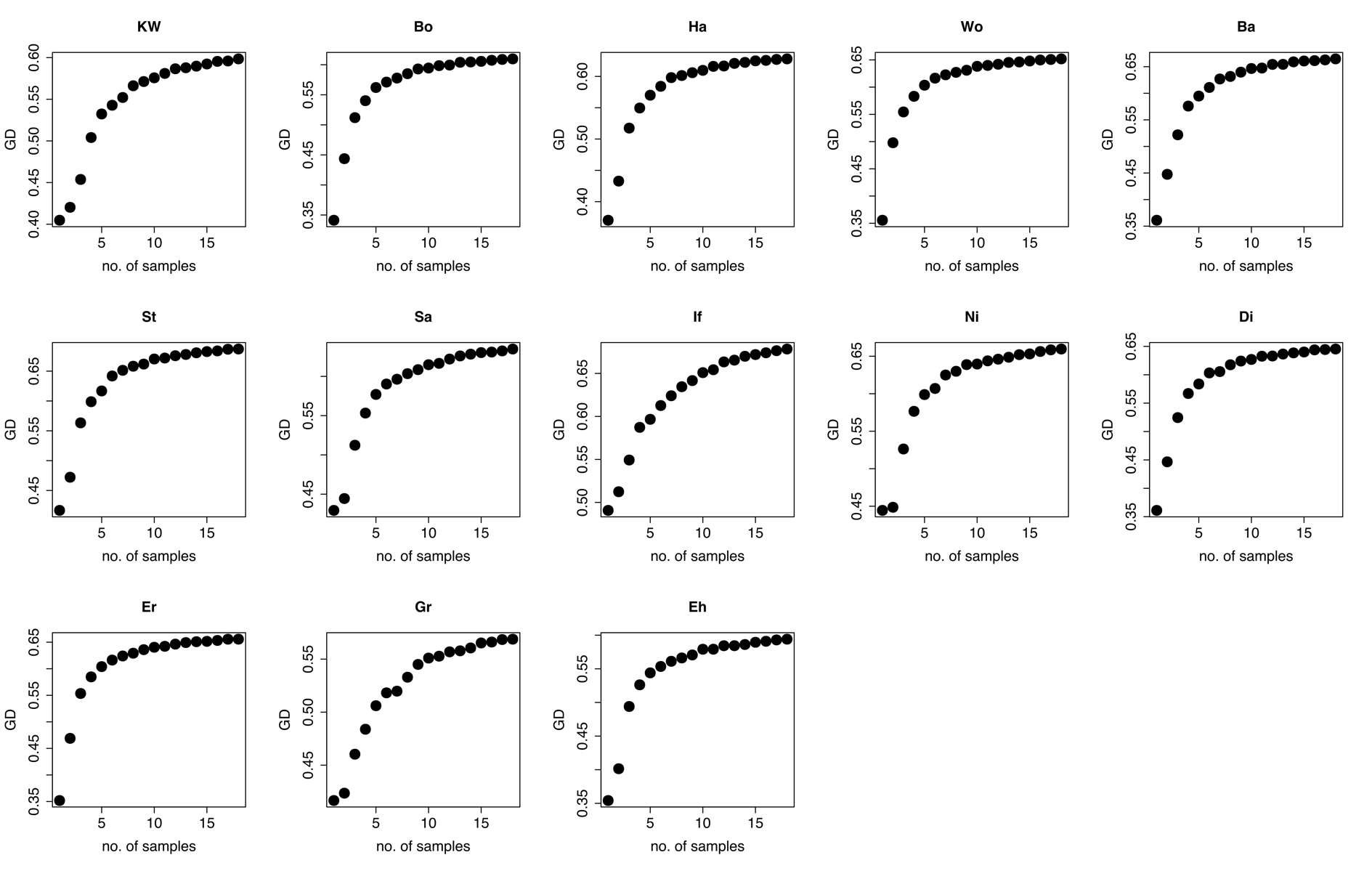


**Fig. S8** Genetic diversity (GD, H_e_) per population for different numbers of randomly chosen samples (1-18, with 100 replicates per sample number; see Methods for details).

**Table S2.** Correlation matrix based on Pearson coefficents between coefficients of variation of particular traits (CVtrait) and of particular abiotic environmental factors (CVfactor) in 13 *T. montanum* populations (n = 260 individuals) of Central Europe. We only illustrated (marginally) significant results. See Table 2 for abbreviations and see Fig. 6 for the visualized correlation matrix.

|  | **CV_altitude_** | **CV_slope exposure_** | **CV_slope_** | **CV_LAI_** | **CV_soil depth_** | **CV_CECpot_** | **CV_pH_** | **CV_N_** | **CV_P_** | **CV_K_** |
| --- | --- | --- | --- | --- | --- | --- | --- | --- | --- | --- |
| CV_RH_ |  | r = 0.77, p < 0.01 | r = 0.51, p = 0.07 |  |  |  |  | r = 0.68, p < 0.05 |  |  |
| CV_AGB_ |  | r = 0.60, p < 0.05 | r = 0.57, p < 0.05 |  |  |  |  | r = 0.69, p < 0.01 |  |  |
| CV_LA_ |  | r = 0.49, p = 0.09 |  |  |  |  |  |  |  |  |
| CV_SLA_ |  |  |  |  |  |  |  |  | r = 0.67, p < 0.05 |  |
| CV_LDMC_ |  |  |  |  |  |  |  |  | r = 0.61, p < 0.05 |  |
| CV_Fv/Fm_ |  |  |  |  |  |  |  |  |  | r = 0.54, p = 0.06 |
| CV_PI_ |  |  | r = 0.53, p = 0.06 |  |  |  |  |  |  | 0.55, p = 0.05 |
| CV_SPS_ |  |  |  |  |  |  |  |  |  |  |
| CV_SPI_ |  | r = 0.53, p = 0.06 |  |  |  |  |  |  |  |  |

**Table S3.** Distance matrix of studied locations (in kilometres, km). Mean distance = 133 km (80 km without KW), standard deviation = 124 km (41 km without KW). Distances were calculated with the R package ‘geosphere’ vers. 1.5-5 (Hijmans, 2016). Distances were calculated with the ‘Vincenty (ellipsoid)’ method that is very accurate but computationally more intensive.

|  | lon | lat | KW | Bo | Ha | Wo | Ba | St | Sa | If | Ni | Di | Er | Gr | Eh |
| --- | --- | --- | --- | --- | --- | --- | --- | --- | --- | --- | --- | --- | --- | --- | --- |
| Riezlern (KW) | 10.173825 | 47.361036 | - | 453 | 439 | 423 | 450 | 452 | 382 | 408 | 385 | 424 | 351 | 368 | 346 |
| Bottendorf (Bo) | 11.396525 | 51.316042 | 453 | - | 18 | 52 | 33 | 45 | 75 | 140 | 72 | 33 | 160 | 86 | 122 |
| Hardisleben (Ha) | 11.446789 | 51.162917 | 439 | 18 | - | 36 | 44 | 56 | 58 | 144 | 63 | 30 | 157 | 71 | 114 |
| Jena-Wogau (Wo) | 11.665083 | 50.924306 | 423 | 52 | 36 | - | 79 | 90 | 45 | 169 | 72 | 56 | 171 | 59 | 121 |
| Bad Frankenhausen (Ba) | 11.103056 | 51.367267 | 450 | 33 | 44 | 79 | - | 12 | 86 | 110 | 65 | 31 | 137 | 94 | 109 |
| Steinthaleben (St) | 11.00485 | 51.40955 | 452 | 45 | 56 | 90 | 12 | - | 95 | 101 | 69 | 40 | 132 | 101 | 108 |
| Saalfeld (Sa) | 11.383729 | 50.631003 | 382 | 75 | 58 | 45 | 86 | 95 | - | 145 | 42 | 55 | 136 | 15 | 81 |
| Ifta (If) | 10.148017 | 51.086633 | 408 | 140 | 144 | 169 | 110 | 101 | 145 | - | 103 | 115 | 57 | 141 | 88 |
| Niederwillingen (Ni) | 11.027711 | 50.776294 | 385 | 72 | 63 | 72 | 65 | 69 | 42 | 103 | - | 39 | 99 | 40 | 52 |
| Dielsdorf (Di) | 11.188406 | 51.095233 | 424 | 33 | 30 | 56 | 31 | 40 | 55 | 115 | 39 | - | 128 | 63 | 89 |
| Erbenhausen (Er) | 10.157383 | 50.565556 | 351 | 160 | 157 | 171 | 137 | 132 | 136 | 57 | 99 | 128 | - | 126 | 57 |
| Großneundorf (Gr) | 11.294961 | 50.532456 | 368 | 86 | 71 | 59 | 94 | 101 | 15 | 141 | 40 | 63 | 126 | - | 70 |
| Ehrenberg (Eh) | 10.665786 | 50.478583 | 346 | 122 | 114 | 121 | 109 | 108 | 81 | 88 | 52 | 89 | 57 | 70 | - |
